# A novel signalling screen demonstrates that CALR mutations activate essential MAPK signalling and facilitate megakaryocyte differentiation

**DOI:** 10.1038/leu.2016.280

**Published:** 2016-12-02

**Authors:** K Kollmann, W Warsch, C Gonzalez-Arias, F L Nice, E Avezov, J Milburn, J Li, D Dimitropoulou, S Biddie, M Wang, E Poynton, M Colzani, M R Tijssen, S Anand, U McDermott, B Huntly, T Green

**Affiliations:** 1 Cambridge Institute for Medical Research and Wellcome Trust/MRC Stem Cell Institute, University of Cambridge, Cambridge, UK; 2 Department of Haematology, University of Cambridge, Cambridge, UK; 3 Cambridge Institute for Medical Research, Wellcome Trust MRC Institute of Metabolic Science and NIHR Cambridge Biomedical Research Centre, Cambridge, UK; 4 Department of Haematology, University of Cambridge, and National Health Service Blood and Transplant, Cambridge Biomedical Campus, Cambridge, UK; 5 Cancer Genome Project, Wellcome Trust Sanger Institute, Genome Campus, Hinxton, Cambridgeshire, UK; 6 Cambridge University Hospitals NHS Foundation Trust, Cambridge, UK

## Abstract

Most myeloproliferative neoplasm (MPN) patients lacking JAK2 mutations harbour somatic CALR mutations that are thought to activate cytokine signalling although the mechanism is unclear. To identify kinases important for survival of *CALR*-mutant cells, we developed a novel strategy (KISMET) that utilizes the full range of kinase selectivity data available from each inhibitor and thus takes advantage of off-target noise that limits conventional small-interfering RNA or inhibitor screens. KISMET successfully identified known essential kinases in haematopoietic and non-haematopoietic cell lines and identified the mitogen activated protein kinase (MAPK) pathway as required for growth of the *CALR*-mutated MARIMO cells. Expression of mutant CALR in murine or human haematopoietic cell lines was accompanied by myeloproliferative leukemia protein (MPL)-dependent activation of MAPK signalling, and MPN patients with *CALR* mutations showed increased MAPK activity in CD34 cells, platelets and megakaryocytes. Although *CALR* mutations resulted in protein instability and proteosomal degradation, mutant CALR was able to enhance megakaryopoiesis and pro-platelet production from human CD34^+^ progenitors. These data link aberrant MAPK activation to the MPN phenotype and identify it as a potential therapeutic target in *CALR*-mutant positive MPNs.

## Introduction

In 2005, a single recurrent gain-of-function mutation in the JAK2 tyrosine kinase (*JAK2V617F*) was discovered in the majority of patients with myeloproliferative neoplasms (MPNs),^1, 2, 3, 4^ with this discovery allowing precise diagnosis and catalysing the development of several therapeutic JAK2 inhibitors. In 2013, somatic *CALR* mutations were identified in most *JAK2*-unmutated patients with essential thrombocythaemia or primary myelofibrosis patients.^5, 6^ Multiple *CALR* mutations that generated a +1 bp frameshift and resulted in mutant proteins with a novel C-terminus were demonstrated in exon 9. One insertion (K385fs*47) and one deletion (L367fs*46) mutation were particularly common (together constituting 85% of *CALR* mutations), with these separate mutations exhibiting different disease associations. Several lines of evidence, such as the consistent generation of a novel C-terminus and the lack of truncating mutation, indicate that mutations are gain-of-function (for example, consistent generation of novel C-terminus; lack of truncating mutations).

CALR is ubiquitously expressed and normally resides in the endoplasmic reticulum (ER), where it ensures proper glycoprotein folding and also contributes to calcium storage and modulation of calcium homoeostasis.^7, 8^ In addition, CALR functions outside the ER, at the cell surface and in the extracellular matrix, where it is described to modulate cellular processes, including adhesion, blood function, gene expression and phagocytosis.^9, 10, 11, 12^ However, the cellular and biochemical consequences of *CALR* mutations remain largely unknown. CALR mutations and JAK2/myeloproliferative leukemia protein (MPL) mutations are almost completely mutually exclusive in MPN patients, suggesting that mutant CALR also activates cytokine signalling. In support of this, ectopic expression of mutant *CALR* in interleukin-3 (IL3)-dependent murine Ba/F3 cells conferred (MPL)-dependent increased JAK/STAT phosphorylation together with cytokine-independent growth,^13^ and expression profiling of granulocytes from patients with *JAK2*-mutant or *CALR*-mutant MPNs suggested many similarities.^14^ However, analysis of direct STAT targets from primary megakaryocytes revealed substantial differences in STAT signalling downstream of JAK2 and CALR mutations,^15^ and CALR-mutant MARIMO cells lack active JAK–STAT signalling and are unresponsive to JAK2 inhibitors.^16^

Many kinases play key roles in multiple pathways downstream of cytokine signalling. The human genome encodes 518 protein kinases,^17^ the dysregulation of which is associated with a wide range of human diseases.^18^ A rewiring of cellular signalling networks underlies the development and evolution of many forms of cancer, with multiple kinase pathways often corrupted within a single tumour. Although there are multiple examples of classical oncogene dependence with a single kinase representing a point of vulnerability,^19^ it is frequently difficult to predict exactly which kinases most strongly contribute to tumour pathogenesis. Functional *in vitro* screens have been widely used to address this challenge, and include the use of libraries of small-interfering RNA (siRNA) constructs or small-molecule inhibitors.^20, 21^ However such screens often generate large numbers of false-positive hits, forcing researchers to allocate significant resources to validation and follow-up studies of each potential candidate kinase. The most problematic source of false-positive results are ‘off-target' effects and much effort has been spent trying to reduce this background noise.

Here we report a novel approach that turns off-target noise to our advantage. KISMET (Kinase Inhibitor Screen for Mapping Essential Targets) provides a reliable and inexpensive method for identifying essential kinases, and identified the mitogen activated protein kinase (MAPK) pathway as essential for CALR-mutant MARIMO cells. We demonstrate that mutant CALR, although unstable and readily degraded in a proteasome-dependent manner, activates MAPK signalling and triggers enhanced megakaryocytic differentiation.

## Materials and methods

### Cell lines, infections and transient transfections

Marimo, K562, HEL, UKE-1, SET-2, HL-60, Dami, Ba/F3 and 32D cells were cultured in RPMI (Sigma, St Louis, MO, USA), 10% fetal calf serum (Life Technologies, Waltham, MA, USA) and penicillin/streptomycin (100 U/ml, 100 mg/ml). UKE-1 cells were cultured in 20% fetal calf serum. HEK293T (293T) were cultured in Dulbecco's modified Eagle's medium (Sigma), 10% fetal calf serum (Life Technologies) and penicillin/streptomycin (100 U/ml, 100 mg/ml). Human wild-type CALR and mutant CALR insertion (K385fs*47) and deletion (L367fs*46) cDNA, alone or fused to FLAG or FLAG-mCherry, were cloned into the pCDF1-MSC2-EF2-copGFP lentiviral vector (System Biosciences, Palo Alto, CA, USA) or the pCCL-PPT-MNDU3-PGK-GFP lentiviral vector^22^ and sequence-verified. In addition, all constructs carrying a FLAG or FLAG-mcherry had a signal peptide site at their N-terminus, enabling CALR to enter the endoplasmatic reticulum. Lentivirus was produced by transient transfection of 293T cells and concentrated with Lenti-X concentrator (Clontech, Saint-Germain-en-Laye, France). Cell lines have been infected with concentrated lentivirus (multiplicity of infection of 20, as titered on HEK293T cells) with 8 μg/ml polybrene for 12 h prior to washing and were sorted for GFP expression 24 h after infection. Human CD34^+^ cell-enriched populations from cord blood (>90% pure) were isolated by immunomagnetic selection with the CD34 Microbead Kit (Miltenyi Biotec, Bergisch Gladbach, Germany). Isolated cells were cultured in a density of 1 × 10^5^ cells/ml in SCGM (CellGenix, Freiburg im Breisgau, Germany) with 100 ng/ml hTPO and 10 ng/ml hIL-1β. After 2 days cells have been exposed to concentrated lentivirus (multiplicity of infection of 50, as titered on HEK293T cells) with 8 μg/ml polybrene for 12 h prior to washing and were sorted for GFP expression 24 h after infection. 293T cells have been transiently transfected with Turbofect (Life Technologies) according to the manufacturer's protocol.

### Western blots and co-immunoprecipitation

Cell lysates were made and immunoblotting was performed as described previously.^23, 24^ Antibodies used during the study were CALR (Millipore, Darmstadt, Germany), HSC-70 and β-actin (Santa Cruz, Dallas, TX, USA), ERK1/2, pERK1/2, MEK1/2, pMEK1/2 (all Cell Signaling, Danvers, MA, USA) and FLAG (Abcam, Cambridge, UK). For co-immunoprecipitations 1000 μg of cell lysates were incubated with Anti-FLAG M2 Magnetic Beads (M8823; Sigma) and protein has been immunoprecipitated according to the manufacturer's protocol. Samples were heated for 5 min at 95 °C to separate beads from proteins. The reaction mixtures were run on a sodium dodecyl sulphate polyacrylamide gel.

### Real-time PCR and and RAS–RAF sequencing

For real-time PCR RNA was isolated using TriZol (Invitrogen, Carlsbad, CA, USA). First-strand cDNA synthesis and PCR-amplification were performed using the Tetro cDNA FAST qPCR Master Mix (Bioline, London, UK) according to the manufacturer's instructions. qPCR was performed using KAPA SYBR FAST qPCR Master Mix (KAPA Biosystem, Waltham, MA, USA). The following primer sequences were used: *CALR* F: GAGCCTGCCGTCTACTTCA and R: AACTGAGAACGAATTTGCCA. Each experiment was performed in duplicates and results were normalized by comparison with *RPLPO* mRNA expression.

For RAS–RAF sequencing cDNA synthesis was carried out using Tetro cDNA synthesis kit (Bioline) according to the manufacturer's instructions. PCR was carried out using AmpliTaq Gold 360 PCR Master Mix (Thermo Fisher, Wilmington, MA, USA). Ten cycles of touchdown PCR from 68 to 58 °C followed by 30 cycles at 58 °C with extension times of 2 min were used for amplification according to the manufacturer's instructions. The primers used for sequencing are shown in Supplementary Table S3.

Following primers were used to sequence RAF and RAS genes: ARAF1 F: AAGAGAGGCCCAAGATGGAG, R: CTGAGATTGGCTCCCTGGT; ARAF2 F: ACCTCAGCCCATCTTGACAA, R: GTCCCGATCCTCTTCAGCAG, BRAF1 F: GGCTCTCGGTTATAAGATGGC, R: CGGAACAGAAAGTAAAGCCT CT; BRAF2 F: ATACCACAGGAAGAGGCGTC, R: GCACATTGGGAGCTGATGAG; CRAF1 F: CCTGGCTCC CTCAGGTTTAA, R: ACATGTGTTCTGCCTCTGGA; CRAF2 F: GGAGTCCCAGCACTACCTTC, R: ATCCTGCA TTCGGATCACCT; HRAS F: CTGTGAACGGTGGGGCAG, R: ACTGTGATCCCATCTGTGCC; KRAS F: ATTTCGG ACTGGGAGCGAG, R: AGAATCATCATCAGGAAGCCC; N-RAS F: CCGCATGACTCGTGGTTC, R: AGGGAGTA ACAAGAGGTGCA.

### Flow cytometric analysis and intracellular FACS staining

Transient transfected 293T cells were analysed for GFP and mCherry expression (MFI and %) over time. For megakaryocytic differentiation of infected CD34^+^ cord blood cells, CD41 (APC-Cy7 conjugated; Biolegend, San Diego, CA, USA) and CD42a (APC conjugated; Miltenyi Biotec)-positive cells were determined after 8 days of hTPO and hIL-1ß treatment. Percent of GFP-expressing infected cord blood cells have been analysed over time for cell growth. All experiments have been analysed by a Becton Dickinson (BD) Fortessa LSR II and data were analysed in Flowjo (FLowJo, LLC, Ashland, OR, USA). Intracellular FACS staining has been performed as described before^25^ using antibodies against pMEK1/2 (PE Mouse anti-MEK1 (pS218)/MEK2 (pS222); BD) and pSTAT5 (Alexa Fluor 647 Mouse Anti-Stat5 (pY694); BD).

### Pro-platelet forming assay, platelet isolation, purity measurement and differentiation of patient CD34^+^ cells

After 8 days of differentiation, infected cord blood CD34^+^ cells were counted and the same number of cells was plated in SCGM (CellGenix) on fibrinogen-coated (200 mg/ml) 96-well plates. After 4–7days the assay was analysed. The percentage of pro-platelet-forming megakaryocytes have been counted against the total number of megakaryocytes adhering to the surface.^26^

Platelets from patients and healthy volunteers have been isolated, lysed in sodium dodecyl sulphate sample buffer and prepared for western blot as described previously.^23, 27^ Purity of platelets have been measured using an ABC VET hematology analyser (Scil Animal Care, Gurnee, IL, USA). Patient progenitor cell-enriched populations from peripheral blood were sorted for CD34 expression using a CD34 antibody (PE conjugated; BD Bioscience). Sorted cells were cultured in a density of 1 × 10^5^ cells/ml in SCGM (CellGenix) with 100 ng/ml hTPO and 10 ng/ml hIL-1β. After 10 days cells were analysed for CD41 and CD42a expression and immediately lysed with sodium dodecyl sulphate sample buffer.^23^

### Microscopy

Prior to immunofluorescence staining cells were fixed with 4% paraformaldehyde, permeabilized with 0.5% Triton X-100/phosphate-buffered saline and blocked with 10% goat serum/phosphate-buffered saline. To visualize the ER chicken anti Bip IgY^28^ was used as primary and goat anti-chicken IgG conjugated to Alexa647 (Life Technologies) as secondary antibodies. FLAG-tagged CALR variants were detected using mouse monoclonal anti-FLAG as primary (Sigma-Aldrich, St Louis, MO, USA), and goat anti mouse IgG conjugated to Alexa Fluor488 (Jackson Immuno Research Laboratories, West Grove, PA, USA) as secondary antibodies. Nuclei were counterstained with Hoechst 33342 (2μg/ml in phosphate-buffered saline). The stained samples were analysed by the laser scanning confocal microscopy system (LSM 780; Carl Zeiss, Jena, Germany) with a Plan-Apo-chromat × 60 oil immersion lens (NA 1.4).

Materials and methods on the KISMET method (cell lines, kinase inhibitors, statistics, single-dose–response values, combination of two off-target data sets, selection of kinases used for KISMET and algorithm for the calculation of the kinase rank) can be found in the Supplementary Information.

## Results

### Development of a drug on- and off-target based screen to identify essential kinases

In order to identify signalling pathways essential for cells harbouring CALR mutations, we developed a novel kinase inhibitor screen that utilizes kinase selectivity data for each inhibitor and thus takes advantage of their off-target profiles (KISMET, kinase inhibitor screen for mapping essential targets). A key aim was to establish an approach that would be of broad utility for the identification of essential kinases required in a variety of transformed cellular contexts. The kinase selectivity profile of more than 200 kinase inhibitors for over 85% of the human kinome has recently been reported (Supplementary Data Set S1).^29, 30^ Although most kinases lacked a highly specific inhibitor, each kinase was inhibited by off-target activities of several inhibitors (Supplementary Figures S1A and B). To establish the KISMET assay we included 279 kinases, showing the strongest affinity for 202 inhibitors (see Materials and methods for details). To increase speed and reduce cost, it was decided to utilize a single concentration of each inhibitor. The aim was to use a dosage that would cause the majority of inhibitors to affect most cell lines, without killing all cells within 72 h. Preliminary experiments using an inhibitor concentration range of 1–5 μm demonstrated that a concentration of 4 μm maximized the heterogeneity of the response of the cell lines to a panel of 70 inhibitors. Using this concentration of 4 μm for all 202 inhibitors against a panel of 44 cell lines, the mean cell survival at 72 h was 66% with 152 inhibitors inducing an average cell death of more than 10% but only four inhibitors causing more than 90% cell death.

**Figure 1:**
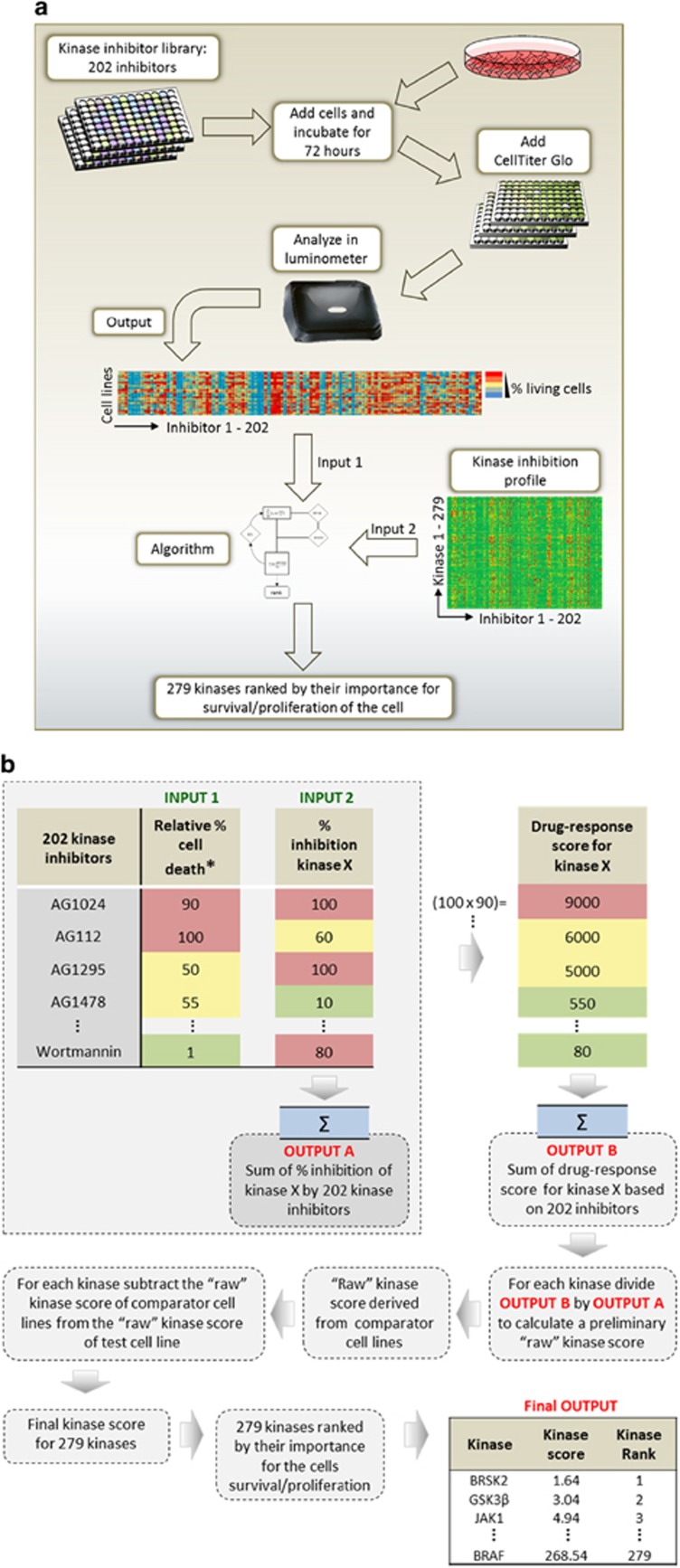
Development of KISMET to screen for essential kinases in cancer cell lines. (**a**) Work flow to generate dose–response data of cancer cells treated with 202 different kinase inhibitors. Dose–response data and the detailed inhibitor off-target profile are integrated into an algorithm which outputs a score for each of the 279 kinases, enabling them to be ranked according to their importance for the survival and/or proliferation of the cells. (**b**) A simplified flowchart of the algorithm is shown.

The method is outlined in Figure 1a. Cells of interest were added to the kinase inhibitors in 96-well plates and the number of viable cells was assessed after 72 h. An algorithm was developed to integrate these data (Input 1; Figure 1b) with the kinase selectivity profiles of the 202 inhibitors (Input 2; Figure 1b) and thus generate a score for each kinase, enabling them to be ranked according to their importance for survival and/or proliferation of the test cells (see Materials and methods for details). Results for each test cell line were related to a panel of comparator cell lines, thus allowing discrimination between cell-selective targets and those that are essential for most cell types. Two different comparator panels were used: one composed of 29 haematopoietic cell lines (panel A), which was used when a hematopoietic cell line was screened, and a second composed of 15 non-hematopoietic cell lines (panel B), which was used for each non-hematopoietic test cell line (Supplementary Figure S1C). A detailed description of KISMET and the algorithm can be found in the Materials and methods section of the Supplementary Information. The final output of KISMET for a given cell line is a score for each kinase which allows the kinases to be ranked for that cell line. The closer a kinase score is to 1.0 the more ‘important' the kinase is for the test cells.

### KISMET reveals a highly active MAPK pathway in CALR-mutant MARIMO cells

**Figure 2:**
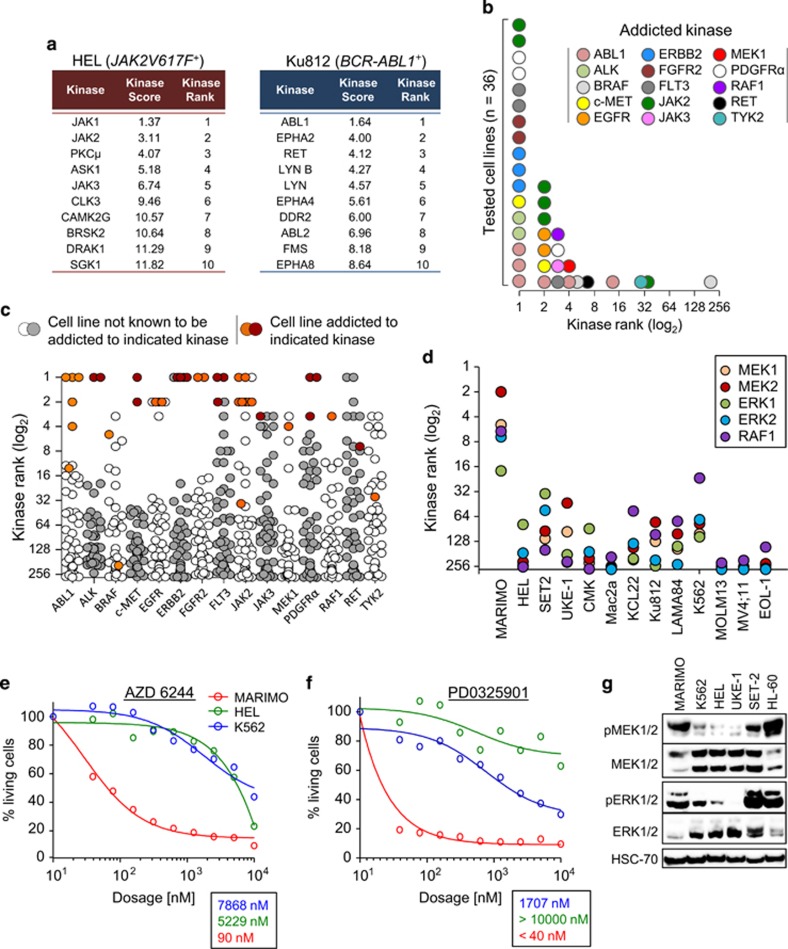
A screen of cancer cell lines with known kinase addictions proves high reliability and robustness of KISMET. (**a**) List of top 10 kinases as ranked by the algorithm for the JAK2-dependent cell line HEL and the BCR-ABL1-dependent cell line Ku812. (**b**) Thirty-six cell lines with known kinase addictions (based on inhibitor studies and/or RNAi mediated knock down of the respective kinase) were screened with KISMET. HL-60 and EOL-1 have been included twice since they have been shown to be addicted to two kinases. The graph shows the rank of the essential kinase for each cell line, as calculated by the algorithm. (**c**) Each column is composed of 44 dots, each of which represents the rank of the kinase indicated below for one of 44 screened cell lines. Orange or red coloured dots represent the kinase ranks of cell lines known to be addicted to the indicated kinase; white and grey dots depict kinase ranks of cell lines not known to be addicted to the indicated kinase. (**d**) The CALR-mutant cell line MARIMO has been screened with KISMET. Shown are the kinase ranks for the MAPK members MEK1, MEK2, ERK1, ERK2 and RAF1 in MARIMO cells as well as 12 other human myeloid cell lines. (**e**, **f**) MARIMO, HEL (*JAK2V617F*^+^) and K562 (*BCR-ABL1*^+^) cells have been used to perform 10-point dose–response assays with the MEK1/2 and ERK1/2 inhibitor AZD 6244 (**e**) or the MEK1/2 inhibitor PD0325901 (**f**). The tables below show the _IC50_ values for all three cell lines upon 72 h of treatment. (**g**) The haematopoietic human cell lines UKE-1 and SET-2 (both *JAK2V617F*^+^ MPN), HL-60 (*NRAS Q61L*^+^ AML) as well as MARIMO, HEL and K562 cells have been analysed by western blot for their MEK1/2 and ERK1/2 activation.

To validate KISMET, we initially focused on six cell lines known to be dependent on either the *JAK2V617F* mutation (UKE-1, HEL and SET-2)^31^ or the *BCR-ABL1* fusion (Ku812, K562 and LAMA84).^32^ For HEL and Ku812 the top 10 kinases ranked by their kinase score are shown in Figure 2a. KISMET ranked JAK2 at position 1, 2 and 2 in the three *JAK2V617F* mutant cell lines and ABL1 at position 1 in all three *BCR-ABL1*-positive cell lines (Supplementary Figures S2A and B). The complete kinase ranking of all cell lines is shown in Supplementary Data Set S2.

To further investigate the accuracy and reliability of KISMET, we then studied 34 different cancer cell lines reported to be addicted to a total of 15 distinct kinases (Supplementary Table S1) together with 10 cell lines with no kinase addiction reported. For each cell line with a known kinase addiction, the KISMET assay resulted in the correct addicted kinase being ranked in the top 7 out of 279 kinases in 32 of 36 cases demonstrating that KISMET provides a sensitive method for identifying essential kinases (Figure 2b and Supplementary Table S2; Supplementary Data Set S2; HL-60 and EOL-1 were included twice since they are each addicted to two distinct kinases). Moreover, the essential kinase was ranked highly in addicted cell lines but not in most non-addicted lines (Figure 2c and Supplementary Data Set S2), indicating that KISMET also displays substantial specificity. Indeed, most false-positive results reflected the fact that, since many inhibitors cannot distinguish between closely related kinases, cell lines addicted to one kinase may give rise to a high kinase rank for related family members or for kinases with similar drug selectivity profile. As an example of this JAK3 was ranked highly in JAK2-dependent cell lines, and EGFR (ERBB1) was ranked highly in ERBB2-dependent cell lines.

Overall the known essential kinase had a kinase score of ⩽5.0 in 30 of 36 cell lines with a known kinase addiction (Supplementary Figure S2C), and in each of the 44 screened cell lines KISMET identified ⩽6 kinases (mean±s.d.=3.3±1.3, mode=4) with a kinase score ⩽5.0. These data indicate that further investigation of maximally four candidates would suffice to identify the correct essential kinase in 84% of cases. Having established the utility of KISMET, it was then used to interrogate the CALR-mutant MARIMO cell line. Whereas JAK2 was ranked first or second in all three *JAK2V617F*-positive cell lines, it was ranked at position 215 in MARIMO cells (Supplementary Data Set S2). By contrast four of the top seven kinases were members of the RAF/MEK/ERK–MAPK pathway (MEK2, MEK1, RAF1 and ERK2 at positions 2, 5, 6 and 7, respectively) (Figure 2d). This pattern was unique to MARIMO cells and was not observed in 12 other human myeloid cell lines. Dose–response assays were then performed on MARIMO, HEL and K562 cells using AZD 6244 (an inhibitor of ERK1/2 and MEK1) and PD0325901 (an inhibitor of MEK1/2) which has not been included in the KISMET assay. MARIMO cells were extremely sensitive, demonstrating low _IC50_ values for both inhibitors compared with the control cell lines (Figures 2e and f). Consistent with these results, western blot analysis demonstrated substantial levels of phosphorylated MEK1/2 and ERK1/2 in MARIMO cells (Figure 2g). Together these data show that MARIMO cells harbour an active MAPK pathway which is essential for their growth. To exclude whether mutations in RAF or RAS genes are responsible for the observed hyper-activation of the MAPK pathway, we sequenced the genes A-, B- and C-RAF as well as K-, H- and N-RAS. No changes were detected except a silent mutation in the gene N-RAS (g.A54A, p.A11A; Supplementary Figure S2D).

### Mutant *CALR* activates the MAPK pathway and encodes an unstable protein

**Figure 3:**
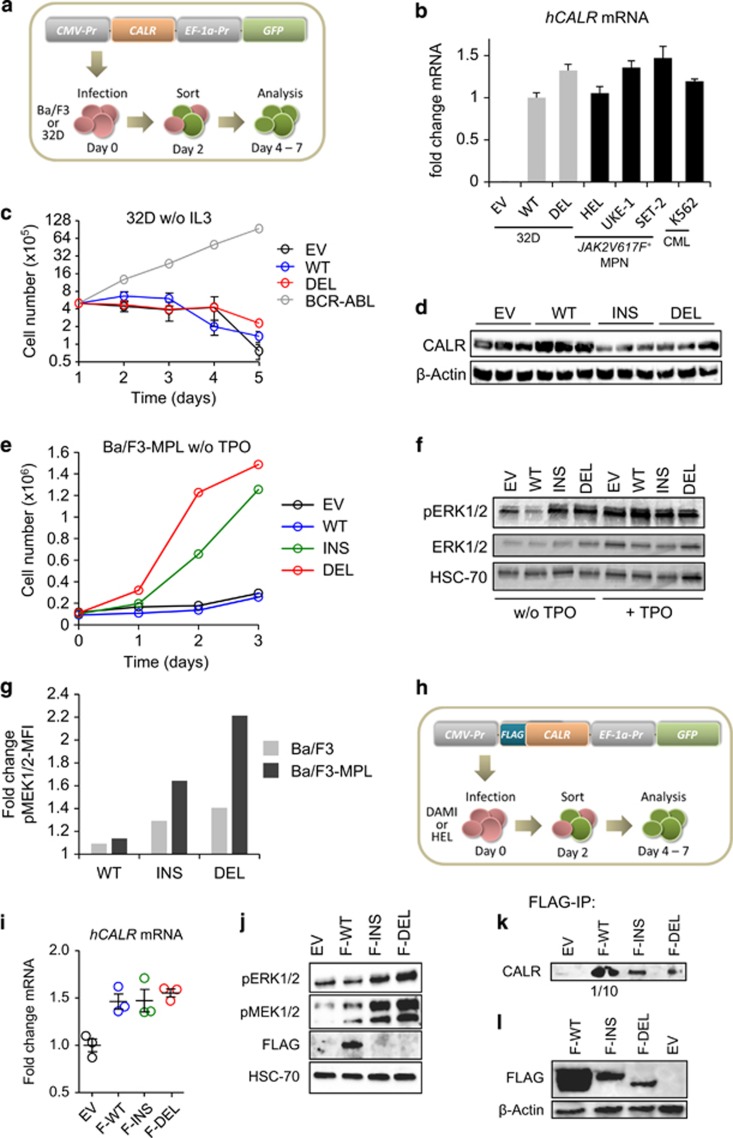
Ectopic expression of mutant CALR enhances MAPK signalling. (**a**) 32D or Ba/F3 cells have been infected with an empty lentivirus (EV) or a lentivirus encoding for human wild-type CALR (WT) or CALR deletion (L367fs*46) mutation (DEL). Two days later cells have been sorted for GFP expression and cultured for another 2–5 days before analysis. (**b**) Real-time PCR of human *CALR* mRNA levels for 32D empty vector (EV), wild-type CALR (WT) and deletion mutant CALR (DEL) cells (*n*=3 cell lines/construct) and four human myeloid cell lines is shown. Human *CALR* mRNA has been normalized to *RPLP0* mRNA. Bar graphs depict the fold change in human *CALR* mRNA compared with 32D WT cells. (**c**) Proliferation assay was performed by plating the same cell numbers of 32D EV, WT and DEL cells (*n*=3 cell lines/construct) without IL3. 32D cells infected with a virus encoding for BCR-ABL served as a positive control. Total cell numbers were determined over 5 days. (**d**) Western blot depicting total CALR protein levels of 32D EV, WT, INS and DEL cells. (**e**) Ba/F3-MPL cells expressing WT or CALR mutants were kept in TPO-free medium and cells were counted for 4 days. (**f**) Ba/F3-MPL cells were infected with a lentivirus encoding for EV, WT or CALR mutants. Shown is a western blot for ERK1/2 and pERK1/2 of cells starved for 4 h (left) and upon 30 min of TPO (10 ng/ml) stimulation (right). (**g**) Parental Ba/F3 and Ba/F3-MPL cells infected with a lentivirus encoding EV, WT or CALR mutants have been starved for 4 h. An intracellular FACS analysis for pMEK1/2 has been performed. The bar graphs depict the fold change in pMEK1/2 mean fluorescence intensity (MFI) compared with EV-control cells. (**h**) Dami or HEL cells have been infected with an empty lentivirus (EV) or a lentivirus encoding for FLAG-CALR wild-type (F-WT), FLAG-CALR insertion (K385fs*47) mutation (F-INS) or FLAG-CALR deletion (L367fs*46) mutation (F-DEL). Two days later cells have been sorted for GFP expression and cultured for another 2–5 days before analysis. (**i**) Real-time PCR for human *CALR* mRNA levels of Dami EV, F-WT, F-INS and F-DEL cells is shown. Human *CALR* mRNA has been normalized to *RPLP0* mRNA. Bar graphs depict the fold change in human *CALR* mRNA compared with Dami EV. The graph depicts data points generated in triplicates of one representative experiment out of two. (**j**) Western blot showing pERK1/2, pMEK1/2 and FLAG protein levels of Dami EV, F-WT, F-INS and F-DEL cells. (**k**) An anti-FLAG co-immunoprecipitation was performed with Dami EV, F-WT, F-INS and F-DEL cell extracts and immunoblotted for total CALR. For F-WT only 1/10 of the precipitate volume compared with EV, F-INS and F-DEL has been loaded. (**l**) 293T cells were transiently transfected with either an EV, F-WT, F-INS or F-DEL construct. After 24 h FLAG protein levels have been analysed by western blot.

MARIMO cells may contain other oncogenic changes and our MARIMO data do not show that CALR mutations play a causal role in activating the MAPK pathway. To investigate whether mutant CALR causes increased MAPK signalling, murine IL3-dependent 32D cells were infected with lentiviral CALR constructs (Figure 3a). Transcript levels of exogenous WT and mutant CALR were similar to each other and to endogenous CALR transcript levels in human haematopoietic cell lines (Figure 3b). In the absence of IL3, both WT and mutant CALR failed to rescue proliferation, in contrast to BCR-ABL (Figure 3c), and we did not observe any activation of ERK1/2 (Supplementary Figure S3A). No altered proliferation was detected in the presence of IL3 (Supplementary Figure S3B). Using an antibody that recognized both murine and human CALR, total CALR protein levels were increased in cells harbouring WT CALR but not mutant CALR, raising the possibility of altered protein translation or degradation (Figure 3d). Similar results were obtained using a second cytokine-dependent murine cell line, Ba/F3 (Supplementary Figures S3C–E).

Since neither 32D nor Ba/F3 cells express MPL, we assessed the consequences of expressing exogenous MPL. Ba/F3-MPL cells harbouring mutant CALR exhibited TPO-independent proliferation (Figure 3e and Supplementary Figure S3F) and showed increased levels of pERK1/2 in the absence of TPO (Figure 3f). Furthermore, intracellular FACS data confirmed that mutant CALR-driven phosphorylation of MEK1/2 depends on the presence of MPL (Figure 3g). We further detected an MPL-dependent activation of STAT5 by mutant CALR, which is consistent with recently published western blot data^13^ (Supplementary Figure S3G). Interestingly, no expression of MPL was observed in MARIMO cells on the mRNA or protein level (Supplementary Figures S3H–I), suggesting that mutant CALR uses an alternative pathway or receptor to activate the MAPK pathway in MARIMO cells.

To extend our analysis of MAPK signalling to human cells with megakaryocytic potential initial experiments were performed using the DAMI cell line.^33^ To monitor protein levels of exogenous CALR, constructs with an N-terminal FLAG tag were generated using a lentiviral vector (Figure 3j). WT and mutant CALR constructs gave rise to equivalent transcript levels (Figure 3k), and both insertion and deletion mutants gave rise to clearly increased levels of pERK1/2 and pMEK1/2 (Figure 3j). Of particular note, an antibody to FLAG detected WT but not mutant CALR fusion proteins (Figure 3j). Similar results were obtained using HEL cells (Supplementary Figures S3H and I), a second cell line that can also undergo megakaryocytic differentiation.^34^ Low levels of mutant CALR could be detected if immunoprecipitation was used to enrich for the FLAG-CALR fusion proteins (Figure 3k) and also by using transient transfection to produce high levels of transfected construct per cell (Figure 3l).

Together these data demonstrate that mutant CALR causes MAPK activation in both murine and human haematopoietic cells, that this is MPL-dependent in the absence of TPO and that mutant CALR constructs generate substantial lower protein levels than their WT equivalents.

### Mutant CALR is rapidly targeted by the proteosomal machinery

**Figure 4:**
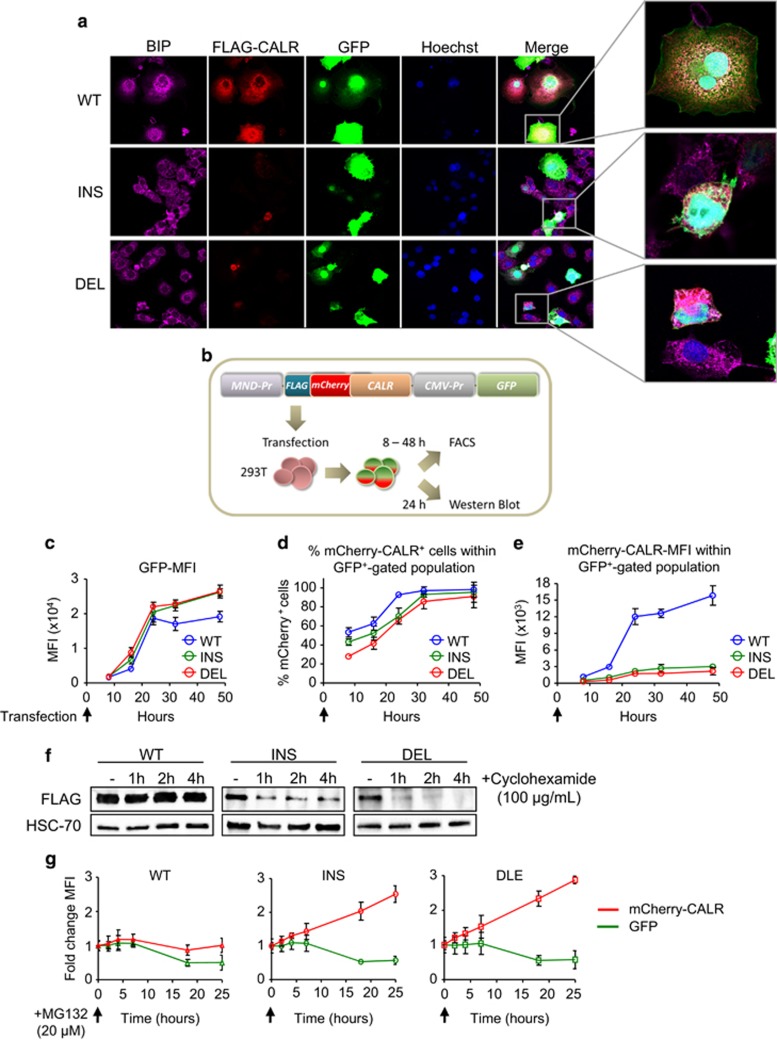
Mutant CALR is rapidly degraded by the proteosomal machinery. (**a**) COS-7 cells have been transfected with FLAG-CALR constructs encoding for GFP on the same vector 24 h prior fixation and microscopy analysis. Cells have been stained with antibodies against BiP, a protein present in the ER (purple) and FLAG (red). (**b**) Scheme: 293T cells have been transiently transfected with either FLAG-mCherry-CALR wild type (mCherry-WT), FLAG-mCherry-CALR insertion (K385fs*47) mutation (mCherry-INS) or FLAG-mCherry-CALR deletion (L367fs*46) mutation (mCherry-DEL) constructs. At 8–48h after transfection cells have been analysed. (**c**–**e**) Mean fluorescence intensity (MFI) of GFP (**c**), %mCherry-CALR+ cells of GFP+ gated population (**d**) and MFI of mCherry-CALR within GFP+ gated population (**e**) have been analysed in 293T mCherry-WT, mCherry-INS and mCherry-DEL cells by FACS 8–48h after transient transfection (*n*=3). (**f**) At 24 h after transient transfection 293T F-WT, F-INS and F-DEL cells were treated with 100 μg/ml cyclohexamide for 1, 2 and 4 h. Western blots show FLAG protein levels for the different time points (one representative experiment out of two is depicted). (**g**) At 24 h after transient transfection 293T mCherry-WT, mCherry-INS and mCherry-DEL cells were treated with 20 μm MG132 and mCherry expression was analysed by FACS over 24 h. GFP expression was used as a control.

We have previously shown that mutant CALR with a C-terminal FLAG tag showed a normal pattern of ER expression.^6^ Since mutant CALR has a novel C-terminal sequence, we considered the possibility that a C-terminal tag might have masked the consequences of the CALR mutations and therefore used constructs with an N-terminal FLAG tag (Figure 3f). Using a FLAG antibody, tagged WT CALR was readily detected in all transfected (GFP positive) cells. By contrast, tagged mutant CALR was only detected in <10% of transfected cells and in those cells it exhibited an abnormal distribution pattern (Figure 4a). To allow more sensitive detection of mutant CALR fusion proteins FLAG-mCherry-CALR fusion constructs were generated and expressed in 293T cells (Figure 4b). Transfection efficiency was similar in cells receiving WT or mutant CALR as indicated by equivalent expression of GFP (Figure 4c), and, the CALR fusion protein was detected in most transfected cells infected with WT or mutant constructs (Figure 4d). However, the level of mCherry-CALR expression was much lower in cells receiving mutant CALR constructs compared with those receiving the WT construct (Figure 4e and Supplementary Figure S4A).

To further explore the low levels of mutant CALR protein, transiently transfected 293T cells were treated with the protein synthesis inhibitor cycloheximide (Figure 4f). Levels of WT CALR were stable whereas mutant CALR levels rapidly declined indicating that mutant CALR protein is less stable than its WT counterpart, a finding that is in line with previously published data.^35^ Cells harbouring CALR fusion constructs were then treated with the proteasome inhibitor MG132 and mCherry-CALR expression was subsequently analysed by FACS. Levels of WT CALR were stable over 24 h after MG132 treatment whereas levels of both insertion and deletion mutants rapidly increased, indicating that the mutant protein instability relates to an increased proteosomal degradation (Figure 4g).

Together these data demonstrate that mutant CALR is unstable and undergoes proteosomal degradation, but is nonetheless able to activate the MAPK pathway in murine and human haematopoietic cells.

### Mutant CALR enhances megakaryopoiesis and pro-platelet formation in human cord blood cells

**Figure 5:**
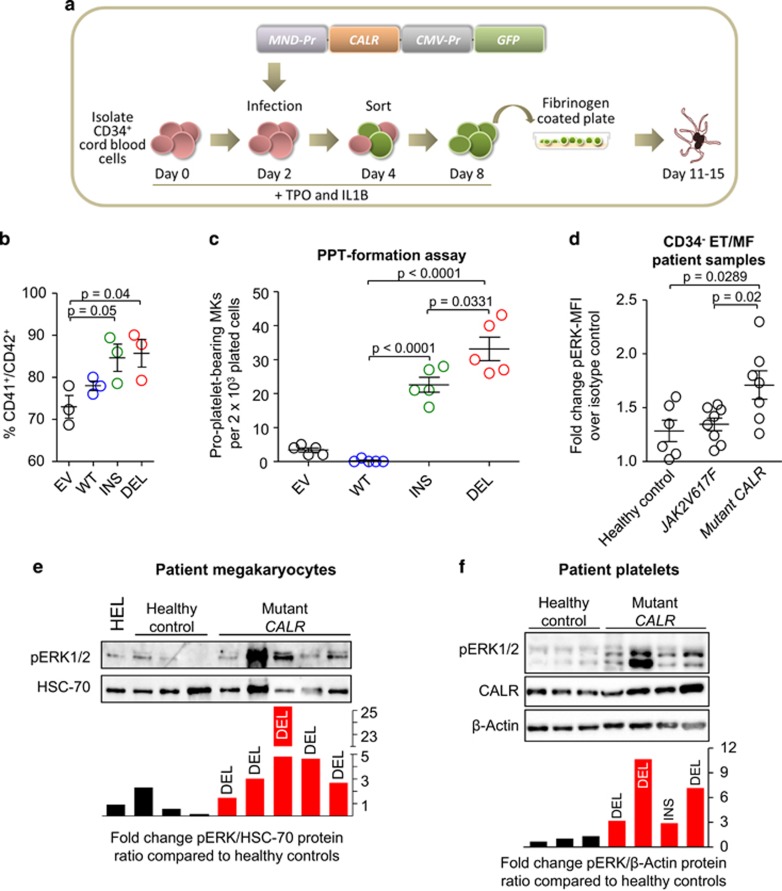
Mutant CALR influences megakaryopoiesis and pro-platelet formation. (**a**) CD34^+^ cells have been isolated from cord blood and differentiated into megakaryocytes in the presence of hTPO and hIL-1β. Two days after the isolation cells have been infected with an empty lentivirus (EV) or a lentivirus encoding for CALR wild type (WT), CALR insertion (K385fs*47) mutation (INS) or CALR deletion (L367fs*46) mutation (DEL). Cells have been sorted for GFP expression and cultured for another 4 days before FACS analysis or performing a pro-platelet formation assay. (**b**) After 8 days of differentiation cells were analysed by FACS for the megakaryocytic surface markers CD41 and CD42. The graph depicts data points generated by three independent experiments. (**c**) To perform a platelet formation assay 2 × 10^3^ CD34^+^ cells expressing EV, WT, INS or DEL have been seeded on fibrinogen-coated plates after 8 days of differentiation. Data points indicate the number of pro-platelet-forming cells per 2 × 10^3^ seeded cells 4 days after seeding. Each data point represents one individually infected sample. (**d**) MFI pERK1/2 levels of CALR-mutant and JAK2-mutant CD34-cell population compared with normal controls by an intracellular FACS assay. (**e**) Peripheral blood-derived CD34^+^ cells from MPN patients or healthy controls were differentiated *in vitro* in the presence of TPO and IL1ß to form CD41^+^CD42^+^ megakaryocytes (84.9–96.7% purity at day 10 of differentiation). Shown is a western blot for pERK1/2. (**f**) Platelets from CALR-mutant and control peripheral blood have been isolated. The contamination with leukocytes upon purification was between 0 and 0.3%. A western blot for pERK1/2 and CALR has been performed and the pERK1/2 to β-actin ratio of CALR patients compared with healthy controls is depicted in the bottom bar graphs.

To explore the biological consequences of CALR mutations in primary cells human CD34^+^ haematopoietic stem and progenitor cells were infected with lentiviral constructs expressing WT and mutant CALR and grown in the presence of TPO and IL1 QUOTE to favour megakaryocytic differentiation (Figure 5a). Compared with WT CALR, expression of mutant CALR did not alter the number of transfected cells and their viability during the differentiation time course (Supplementary Figures S5A and B) but did give rise to an increased proportion of CD41^+^CD42^+^ cells (Figure 5b). Moreover cells harbouring mutant CALR also generated increased levels of pro-platelet formation in four independent experiments (Figure 5c and Supplementary Figures S5C–E). These data are consistent with the megakaryocytic phenotype exhibited by CALR-mutant MPN patients and demonstrate that mutant CALR enhances the formation of megakaryocytes and platelets.

In view of our results demonstrating that mutant CALR can activate MAPK signalling *in vitro*, we next explored whether the MAPK pathway was activated in primary cells from CALR-mutant patients using three complementary approaches. Firstly JAK2-unmutated MPN patients, in whom intracellular signalling proteins had previously been assessed using phopho-flow,^36^ were genotyped for CALR mutations. Compared with equivalent bone marrow-derived populations from healthy controls, CD34^−^ cells from CALR-mutant patients were found to harbour increased levels of pERK1/2 (Figure 5d) with the CD34^+^ population showing a similar trend that did not reach significance (Supplementary Figure S5F). Secondly, peripheral blood CD34^+^ cells from MPN patients or healthy controls were differentiated *in vitro* in the presence of TPO and IL1ß to form CD41^+^CD42^+^ megakaryocytes. Western blotting demonstrated that 4/5 CALR-mutant patients tested had higher levels of pERK1/2 than all four healthy control samples (Figure 5e). Thirdly platelets were purified from CALR-mutant patients and healthy controls. Western blotting showed increased levels of pERK1/2 in platelets from all four CALR-mutant patients compared with healthy controls (Figure 5f).

Together our results demonstrate that MAPK signalling is increased, not only as a consequence of mutant CALR expression *in vitro* but also in primary cells from CALR-mutant patients.

## Discussion

Kinases that contribute to cancer represent potential therapeutic targets. Common screening assays to identify essential kinases have the disadvantage that the candidate can be hidden within the off-target noise. In this manuscript we describe an inexpensive method (KISMET) for identifying essential kinases that takes advantage of this off-target noise. As proof-of-principle, KISMET was used to demonstrate that CALR-mutant MARIMO cells are dependent on MAPK signalling. We go on to show that CALR mutations result in marked protein instability through proteosomal degradation, but are nonetheless able to activate MAPK signalling, and enhance megakaryozytic differentiation.

*In vitro* screens provide a powerful approach to define unexpected points of vulnerability in tumour cells. Two common screening methods used to identify such vulnerabilities are siRNA and small-molecule library screens. Unintended widespread silencing of transcripts due to off-target effects can be a problem in siRNA screens.^37^ It has been shown that the overlap between independently published screens can be very low.^38^ A recent report demonstrated that siRNAs screens are even more prone to off-target effects than already expected.^39^ In a systematic analysis of genome-wide siRNA screens in human cells, it was shown that the observed phenotypes may largely reflect ‘seed'-sequence-based off-target effects. Phenotypes induced by siRNAs correlated with the seed sequences used and not with the intended gene target.

Making use of kinase inhibitor off-target effects has been attempted before^40^ but the lack of a sufficiently high number of inhibitors, comprehensive off-target profiles and/or appropriate algorithms have limited so far the reliability of this approach. By contrast, we demonstrate here that KISMET predicts the correct essential kinase with high precision and reliability.

KISMET has several advantages that sets it apart from current kinase screening options. It is inexpensive, not labour-intensive (just one dosage per inhibitor suffices for a reliable calculation), does not require special facilities (for example, for viral work) and no specific inhibitors are necessary. The latter is an important point since, for many kinases, no specific inhibitor is commercially available. Furthermore, KISMET can be extended to cover the entire kinome repertoire and, through the inclusion of additional inhibitors with known off-target profiles, the reliability of the output increased. Although here we focus on kinase inhibitors, the KISMET algorithm should also be applicable to additional compound classes (for example, phosphatase or G-protein coupled receptor inhibitors) if their off-target profile is known.

We have used KISMET to show that *CALR*-mutant MARIMO cells depend on MAPK signalling. Although no changes in RAF and RAS genes have been found, MARIMO cells may contain other oncogenic changes and our MARIMO data do not show that CALR mutations play a causal role in activating the MAPK pathway. Furthermore, MARIMO cells do not express MPL, suggesting that that mutant CALR uses an alternative pathway or receptor to activate the MAPK pathway in MARIMO cells. This hypothesis is supported by the finding that mutant CALR can also activate the JAK–STAT pathway independently of MPL by using the GCSF receptor.^41^ However, the MARIMO cell line is so far the only known human cell line carrying a CALR mutation and could be helpful for establishing diagnostic tools, one has to be careful when interpreting molecular data. When we expressed mutant CALR in murine and human cells we could show a consequent increase in MEK1/2 and ERK1/2 activation. Moreover, mutant CALR enhanced megakaryocytic differentiation and pro-platelet formation which is in line with and extends the recent demonstration that *CALR* mutants induce thrombocytosis *in vivo*.^13^ These results are also consistent with previous observations that TPO activates MAPK pathway in megakaryocytic cell lines,^42, 43, 44, 45^ and that inhibition of the pathway inhibits megakaryocytic differentiation of CD34^+^ progenitors purified from peripheral blood or cord blood.^46, 47^ Interestingly, MEK inhibition not only reduced megakaryocytic differentiation but also increased erythroid differentiation,^46, 48^ indicating a critical role for the MEK–ERK pathway in regulating the balance between megakaryopoiesis and erythropoiesis. These observations are consistent with the fact that, unlike JAK2 mutations, CALR mutations are found in essential thrombocythaemia and primary myelofibrosis but not in polycythemia vera.^5, 6^

CALR mutations have also been reported to result in enhanced tyrosine phosphorylation of STAT5.^13^ This observation seemed puzzling given that knocking down STAT5 has been shown to increase megakaryocytic differentiation.^49^ However these apparently paradoxical findings may be reconciled by our recent report of an unexpected role for tyrosine unphosphorylated STAT5 (uSTAT5) in repressing megakaryocytic differentiation.^50^ TPO-induced tyrosine phosphorylation of STAT5 resulted in genome-wide redistribution of STAT5 and loss of uSTAT5-mediated repression of a megakaryocytic transcriptional programme. Taken together our data therefore suggest that the consequences of CALR mutations for megakarypoiesis are likely to be mediated by both MAPK and STAT5 pathways.

## Supplementary Material

Supplementary Information

Supplementary Data Set 1

Supplementary Data Set 2

Supplementary Data Set 3

Supplementary Data Set 4
