# Supplementary material for: A novel signalling screen demonstrates that CALR mutations activate essential MAPK signalling and facilitate megakaryocyte differentiation
Source: Leukemia. 2016 Dec 2;31(4):934–44. doi: 10.1038/leu.2016.280 (PMC5383931; doi:10.1038/leu.2016.280)
Supplement: Supplementary Information [file leu2016280x1.pdf]

Supplementary Information to

A novel signalling screen demonstrates that CALR mutations activate essential MAPK signalling and facilitate megakaryocyte differentiation

	Page number
Supplementary Figure and Table Legend	2 – 4
Supplementary Figures	5 – 10
Supplementary Tables	11 – 13
Supplementary References	14 – 15
Supplementary Note 1	16 – 21
Supplementary Note 2	22 – 23
Supplementary Material and Methods	24 – 30

Supplementary Figure and Table Legend

Supplementary Figure 1. (**A**) The blot depicts the % enzymatic inhibition of the kinase KHS by each of the 202 inhibitors used for KISMET Although no specific inhibitor is available for KHS, its activity is inhibited by several inhibitors as an off-target effect (adapted from [1, 2]). (**B**) Diagram showing the enzymatic inhibition of 279 human kinases by 202 distinct inhibitors. Each vertical lane is composed of 202 dots, each dot represents one of the 202 inhibitors. The graph visualizes that most of the 279 kinases are inhibited to more than 50% by several drugs although only for a small fraction of the 279 kinases, a specific inhibitor is present (adapted from [1, 2]). (**C**) Comparator cell line panel A and B. Data ("kinase rank I") derived from both panels have been integrated into the KISMET-algorithm to calculate the kinase rank of test cells.

Supplementary Figure 2. (**A, B**) Bar graphs depict the relative kinase score and kinase rank for each of the 279 kinases of the JAK2 dependent cell lines UKE-1, HEL and SET2 (A) as well as of the BCR-ABL1 dependent cell lines Ku812, K562 and LAMA84 (B). The relative kinase score is defined as the kinase score of the kinase of interest divided by the kinase score of the top ranked kinase. Each kinase is represented by a grey bar, only the addicted kinase is shown in red (JAK2) or blue (ABL1). (**C**) The graph depicts the kinase score for 36 essential kinases. Each square represents one kinase to which the indicated cell line is addicted. Each kinase above the red line has a KS of 5.0 or lower.

Supplementary Figure 3. (A) Western blot of 32D cells infected with a lentivirus encoding for EV, WT or DEL. Cells have been starved for 4 hours before harvesting. Protein levels of pERK1/2 are depicted. (**B**) Proliferation assay was performed by plating same cell numbers of 32D EV, WT and DEL cells (n=3 cell lines/genotype) in medium containing IL3. Total cell numbers were determined over five days. (**C**) Proliferation assay was performed by plating same cell numbers of Ba/F3 EV, WT, DEL and BCR-ABL$^+$ cells (n=3 cell lines/construct) in medium without IL3. Total cell numbers were determined over five days. (**D**) Proliferation assay was performed by plating same cell numbers of Ba/F3 EV, WT and DEL cells (n=3 cell lines/genotype) in medium containing IL3. Total cell numbers

were determined over five days. (**E**) Western blot of Ba/F3 EV, WT and DEL cells for CALR protein levels. (**F**) 5 x 10^4 Ba/F3-MPL cells were seeded in 96 well plates containing medium with increasing concentrations of TPO ranging from 0 – 10 ng/ml. The relative cell density was measured after 24 hours using a cell titer Glo assay. (**G**) Parental Ba/F3 and Ba/F3-MPL cells infected with a lentivirus encoding EV, WT or CALR mutants have been starved for 4 hours. An intracellular FACS analysis for pSTAT5 has been performed. The bar graphs depict the fold change in pSTAT5 (Alexa Fluor 647) mean fluorescence intensity (MFI) compared to EV-control cells. (**H**) Real-time PCR for human *CALR* mRNA levels of HEL EV, F-WT, F-INS and F-DEL cells is shown. Human *CALR* mRNA has been normalized to *RPLP0* mRNA. Bar graphs depict the fold change in human *CALR* mRNA compared to HEL EV. The graph depicts data points generated in triplicates of one representative experiment out of two. (**H**) Western Blot showing pERK1/2, pMEK1/2 and FLAG protein levels of HEL EV, F-WT, F-INS and F-DEL cells.

Supplementary Figure 4. (A) FACS histograms showing mCherry-CALR levels of 293T cells 48 hours after transfection with WT CALR or mutant CALR.

Supplementary Figure 5. (A, B) $CD34^+$ cord blood cells were infected with EV, WT, INS or DEL (all constructs encoding for GFP) and left unsorted. Depicted is the fold change in the percentage of GFP+ cells (A) and in the percentage living cells (B) over a period of seven days. Each infection has been performed in biological triplicates. **(C-E)** To perform a platelet formation assay 1x 10^4 (C and D) or 2 x 10^3 (E) $CD34^+$ cells expressing EV, WT, INS, or DEL have been seeded on fibrinogen coated plates after eight days of differentiation into megakaryocytes. Each data point represents one individually infected sample and indicates the percentage pro-platelet-bearing megakaryocytes (MKs). **(F)** pERK1/2 MFI levels of *CALR*-mutant or *JAK2*-mutant CD34+ cells compared to healthy controls were analyzed by intracellular FACS.

Supplementary Figure 6. The blot depicts for each of 300 kinases the “sum of inhibition” (SOI) (adapted from (1), (2)). The red square encircles 21 kinases with a SOI < 600 which have been

excluded from KISMET. Shown in the top is the formula used to calculate the SOI for each kinase.

Supplementary Table 1. Table of 44 cell lines screened with KISMET. Previously published kinases to which the cells are addicted are shown and colour coded for a better overview.

Supplementary Table 2. 36 cell lines (HL-60 and EOL-1 have been included twice) with previously published kinase addiction have been screened with KISMET. The rank of the addicted kinase for each cell line, as calculated by the algorithm, is shown in the right column. In 47% (17/36 cell lines) the algorithm ranked the correct addicted kinase at position one and in 89% (32/36 cell lines) in a top-7 position.

Supplementary Table 3. 172 inhibitors as published by Anastassiadis et al.[1] and 30 inhibitors as published by Davis et al.[2] have been used for KISMET. To be able to use both datasets using a single measure, the values for the dissociation constant (Kd) published by Davis et al. have been transformed into % kinase inhibition values as shown in the table (adapted from [22]).

Supp. Figure 1

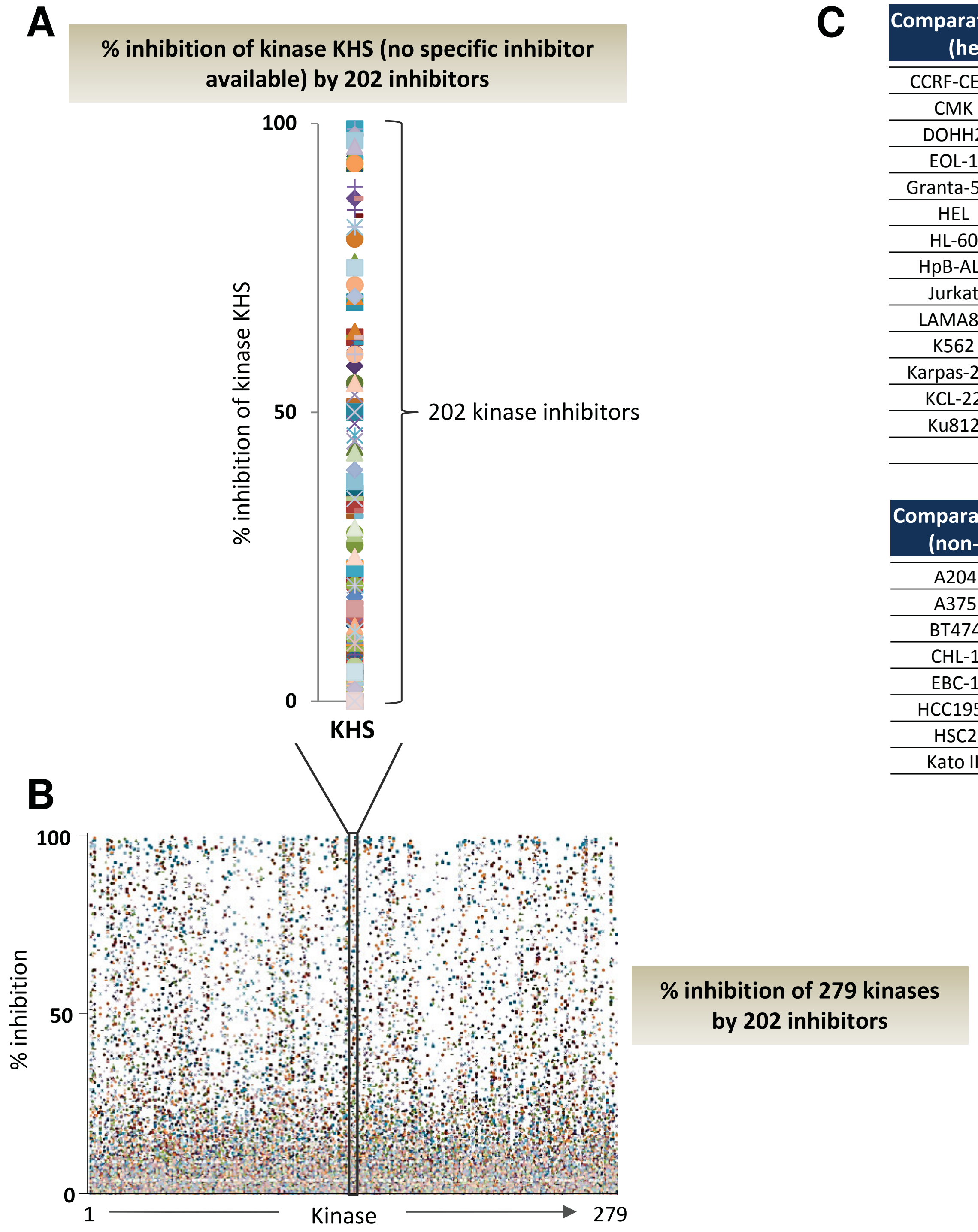

C

Comparator cell line panel A (hematopoietic)	
CCRF-CEM	MOLM13
CMK	MOLT13
DOHH2	MV4;11
EOL-1	PEER
Granta-519	Rec1
HEL	REH
HL-60	SET-2
HpB-ALL	SU-DHL1
Jurkat	UKE-1
LAMA84	Mac2a
K562	32D + IL3
Karpas-299	32D-J2V617F
KCL-22	Ba/F3 + IL3
Ku812	Ba/F3-BCR-ABL1
	Ba/F3-J2V617F

Comparator cell line panel B (non-hematopoietic)	
A204	MCF-7
A375	MFM-223
BT474	MKN45
CHL-1	NCI-H1703
EBC-1	NCI-H358
HCC1954	SK-MEL-28
HSC2	TT
Kato III	

Supp. Figure 2

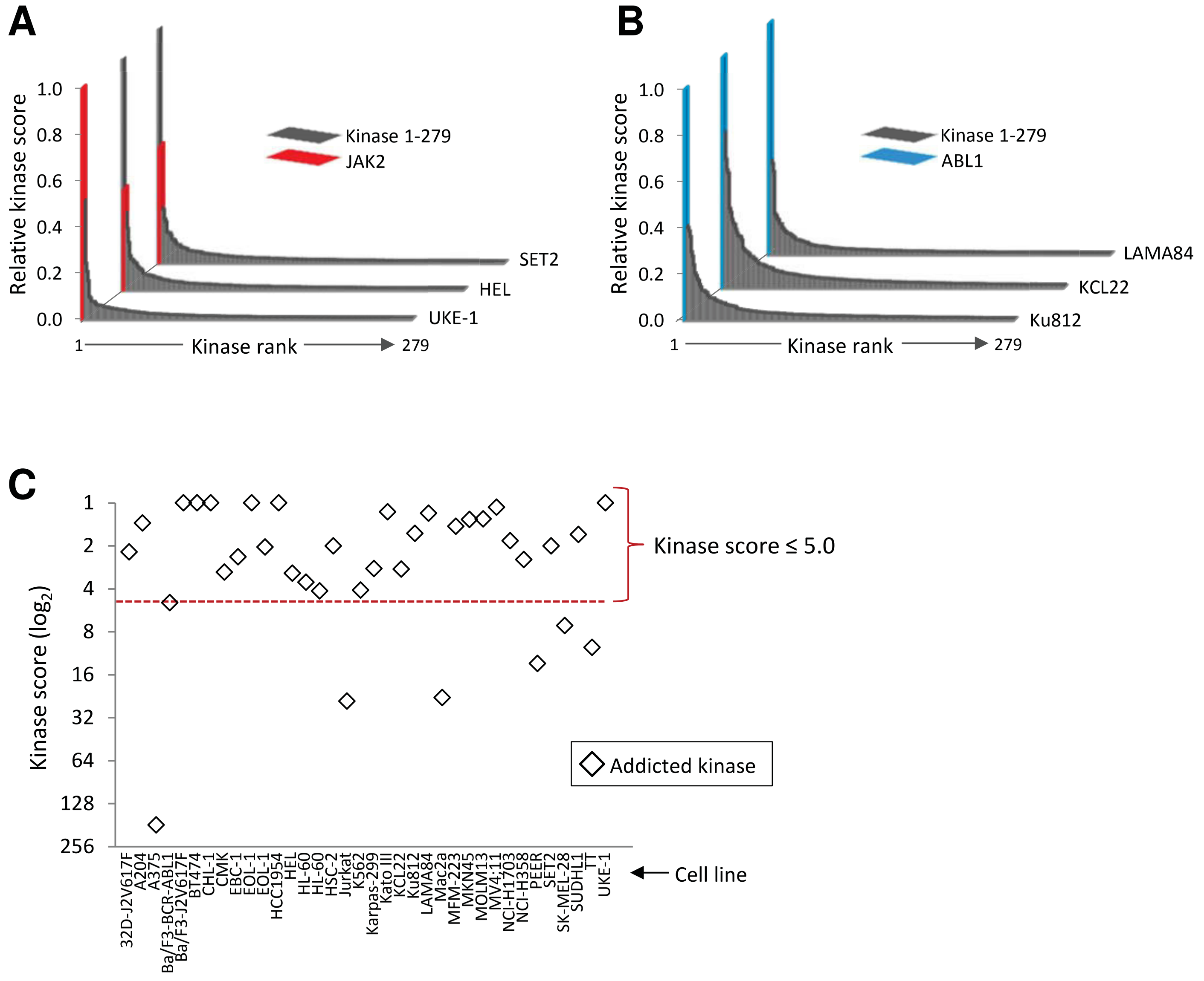

Supp. Figure 3

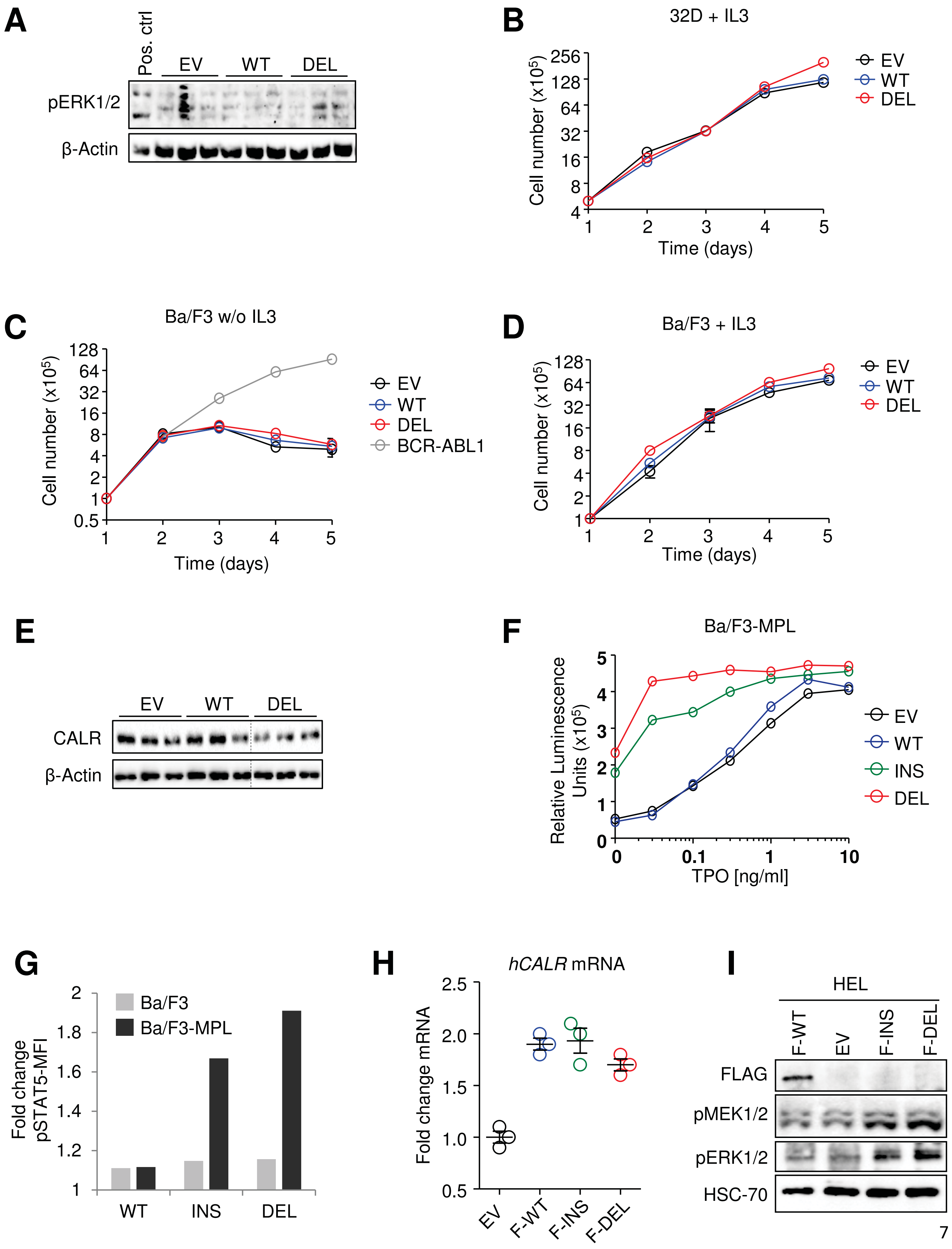

Supp. Figure 4

A

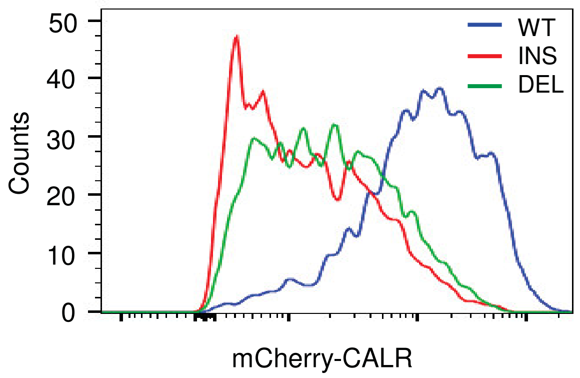

Supp. Figure 5

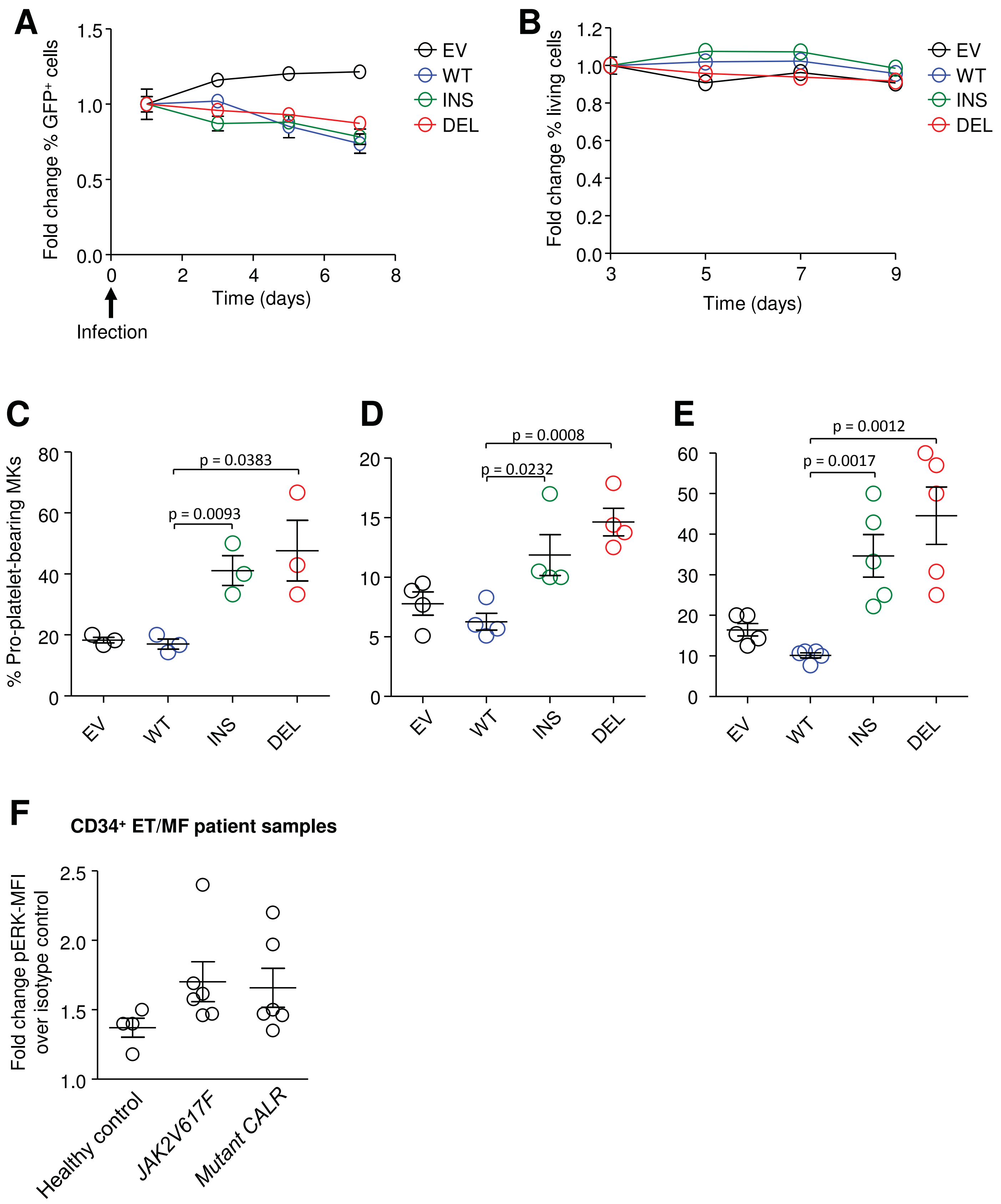

Supp. Figure 6

$$SOI = \sum_{i=1}^{202} \% \; enzymatic\ inhibition\ of\ kinase\ X\ by\ inhibitor\ i$$

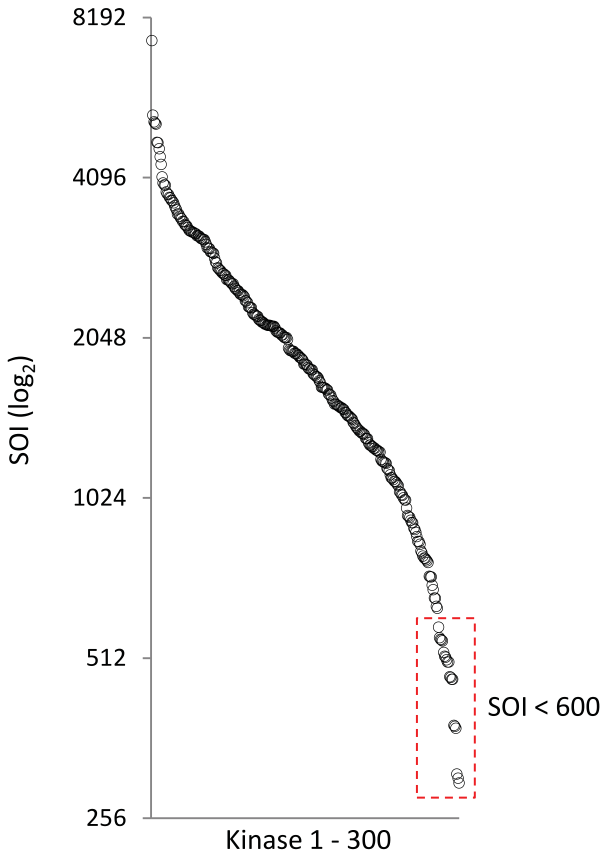

Supp. Table 1

Cell line	Species	Tissue	Origin	Oncogene/ Translocation	Addicted kinase	Ref. kinase addiction
K562	Human	Blood	CML	BCR-ABL1	ABL1	3
KCL-22	Human	Blood	CML	BCR-ABL1	ABL1	3
Ku812	Human	Blood	CML	BCR-ABL1	ABL1	3
LAMA84	Human	Blood	CML	BCR-ABL1	ABL1	3
PEER	Human	Blood	T-ALL	NUP214-ABL1	ABL1	4
Ba/F3-BCR-ABL1	Mouse	Blood	(CML)	BCR-ABL1	ABL1	5
Karpas-299	Human	Blood	Non-Hodgkin lymph., T-cell	NPM-ALK	ALK	6
SU-DHL1	Human	Blood	ALCL	NPM-ALK	ALK	6
MOLM13	Human	Blood	AML	FLT3-ITD	FLT3	7
MV4;11	Human	Blood	AML	FLT3-ITD	FLT3	7
HEL	Human	Blood	MPN	JAKV617F	JAK2	8
Mac2a	Human	Blood	ALCL	PCM1-JAK2	JAK2	9
SET-2	Human	Blood	MPN	JAKV617F	JAK2	8
UKE-1	Human	Blood	MPN	JAKV617F	JAK2	8
32D-J2V617F	Mouse	Blood	(MPN)	JAKV617F	JAK2	10
Ba/F3-J2V617F	Mouse	Blood	(MPN)	JAKV617F	JAK2	11
CMK	Human	Blood	AML	JAK3A572V	JAK3	12
EOL-1	Human	Blood	AML	FIP1L1-PDGFRα	PDGFRα FLT3	13, 14
HL-60	Human	Blood	AML	NRAS Q61L	RAF MEK	15, 15
Jurkat	Human	Blood	T-ALL	-	TYK2	16
CCRF-CEM	Human	Blood	T-ALL	t(5;14)	-	
DOHH2	Human	Blood	B-cell lymphoma	t(14;18)	-	
Granta-519	Human	Blood	Non-Hodgkin lymph., B-cell	t(11;14)	-	
HpB-ALL	Human	Blood	T-ALL	-	-	
MOLT13	Human	Blood	T-ALL	-	-	
Rec1	Human	Blood	Non-Hodgkin lymph., B-cell	t(11;14)	-	
REH	Human	Blood	BCP-B ALL	t(12;21)	-	
32D + IL3	Mouse	Blood	N/A	-	-	
Ba/F3 + IL3	Mouse	Blood	Pro-B cells	-	-	
A375	Human	Skin	Malignant melanoma	BRAFV600E	BRAF	17
SK-MEL-28	Human	Skin	Malignant melanoma	BRAFV600E	BRAF	17
EBC-1	Human	Lung	Carcinoma	c-MET - amplified	c-MET	17
MKN45	Human	Stomach/GI tract	Carcinoma	c-MET - amplified	c-MET	18
HSC2	Human	Upper aerod. tract	Carcinoma	EGFR overexpression	EGFR	19
NCI-H358	Human	Lung	NSCLC: adenocarcinoma	EGFR - autocrine	EGFR	17
BT474	Human	Mammary gland	Breast cancer	HER2 - amplified	ERBB2/HER2	17
HCC1954	Human	Mammary gland	Breast cancer	HER2 - amplified	ERBB2/HER2	17
CHL-1	Human	Skin	Malignant melanoma	HER2 – autocrine	ERBB2/HER2	20
Kato III	Human	Stomach	Carcinoma	FGFR2 - amplified	FGFR2	17
MFM-223	Human	Breast	Carcinoma	FGFR2 - amplified	FGFR2	17
A204	Human	Soft tissue	Rhabdomyosarcoma	PDGFRα - amplified	PDGFRα	17
NCI-H1703	Human	Lung	NSCLC: adenocarcinoma	PDGFRα - amplified	PDGFRα	17
TT	Human	Thyroid	Carcinoma	RETC634W	RET	21
MCF-7	Human	Mammary gland	Breast cancer	-	-	

Supp. Table 2

Cell line	Oncogene	Addicted kinase	**Rank**
Ku812	BCR-ABL1	ABL1	**1**
LAMA84	BCR-ABL1	ABL1	**1**
KCL-22	BCR-ABL1	ABL1	**1**
SU-DHL1	NPM-ALK	ALK	**1**
Karpas-299	NPM-ALK	ALK	**1**
MKN45	c-MET - amplified	c-MET	**1**
BT474	HER2 - amplified	ERBB2/HER2	**1**
HCC1954	HER2 - amplified	ERBB2/HER2	**1**
CHL-1	HER2 – autocrine	ERBB2/HER2	**1**
Kato III	FGFR2 - amplified	FGFR2	**1**
MFM-223	FGFR2 - amplified	FGFR2	**1**
MOLM13	FLT3-ITD	FLT3	**1**
MV4;11	FLT3-ITD	FLT3	**1**
UKE-1	JAKV617F	JAK2	**1**
Ba/F3-J2V617F	JAKV617F	JAK2	**1**
EOL-1	FIP1L1-PDGFRα	PDGFRα	**1**
A204	PDGFRα - amplified	PDGFRα	**1**
Ba/F3-BCR-ABL1	BCR-ABL1	ABL1	**2**
EBC-1	c-MET - amplified	c-MET	**2**
HSC2	EGFR overexpression	EGFR	**2**
NCI-H358	EGFR - autocrine	EGFR	**2**
32D-J2V617F	JAKV617F	JAK2	**2**
SET-2	JAKV617F	JAK2	**2**
HEL	JAKV617F	JAK2	**2**
EOL-1	FIP1L1-PDGFRα	FLT3	**3**
CMK	JAK3A572V	JAK3	**3**
NCI-H1703	PDGFRα - amplified	PDGFRα	**3**
HL-60	NRAS Q61L	RAF1	**3**
K562	BCR-ABL1	ABL1	**4**
HL-60	NRAS Q61L	MEK1	**4**
SK-MEL-28	BRAFV600E	BRAF	**5**
TT	RETC634W	RET	**7**
PEER	NUP214-ABL1	ABL1	13
Jurkat	-	TYK2	29
Mac2a	PCM1-JAK2	JAK2	35
A375	BRAFV600E	BRAF	200

Supp. Table 3

% inhibition	0	5	10	15	20	25	30	35	40	45	50	55	60	65	70	75	80	85	90
Dissociation constant K_d (Davis et al.)	>5000	<5000	<3000	<2000	<1200	<900	<600	<500	<400	<300	<200	<150	<100	<60	<30	<15	<5	<2	<1

Supplementary References

1. Anastassiadis, T., Deacon, S.W., Devarajan, K., Ma, H. & Peterson, J.R. Comprehensive assay of kinase catalytic activity reveals features of kinase inhibitor selectivity. *Nature biotechnology* **29**, 1039-1045 (2011).
2. Davis, M.I. et al. Comprehensive analysis of kinase inhibitor selectivity. *Nature biotechnology* **29**, 1046-1051 (2011).
3. Gambacorti-Passerini, C. et al. Inhibition of the ABL kinase activity blocks the proliferation of BCR/ABL+ leukemic cells and induces apoptosis. *Blood cells, molecules & diseases* **23**, 380-394 (1997).
4. Quintas-Cardama, A. et al. Activity of tyrosine kinase inhibitors against human NUP214-ABL1-positive T cell malignancies. *Leukemia* **22**, 1117-1124 (2008).
5. Redaelli, S. et al. Activity of bosutinib, dasatinib, and nilotinib against 18 imatinib-resistant BCR/ABL mutants. *Journal of clinical oncology : official journal of the American Society of Clinical Oncology* **27**, 469-471 (2009).
6. Hsu, F.Y., Zhao, Y., Anderson, W.F. & Johnston, P.B. Downregulation of NPM-ALK by siRNA causes anaplastic large cell lymphoma cell growth inhibition and augments the anti cancer effects of chemotherapy in vitro. *Cancer investigation* **25**, 240-248 (2007).
7. Yao, Q., Nishiuchi, R., Kitamura, T. & Kersey, J.H. Human leukemias with mutated FLT3 kinase are synergistically sensitive to FLT3 and Hsp90 inhibitors: the key role of the STAT5 signal transduction pathway. *Leukemia* **19**, 1605-1612 (2005).
8. Quentmeier, H., MacLeod, R.A., Zaborski, M. & Drexler, H.G. JAK2 V617F tyrosine kinase mutation in cell lines derived from myeloproliferative disorders. *Leukemia* **20**, 471-476 (2006).
9. Ehrentraut, S. et al. t(8;9)(p22;p24)/PCM1-JAK2 activates SOCS2 and SOCS3 via STAT5. *PloS one* **8**, e53767 (2013).
10. Lu, X. et al. Expression of a homodimeric type I cytokine receptor is required for JAK2V617F-mediated transformation. *Proceedings of the National Academy of Sciences of the United States of America* **102**, 18962-18967 (2005).
11. Pardanani, A. et al. TG101209, a small molecule JAK2-selective kinase inhibitor potently inhibits myeloproliferative disorder-associated JAK2V617F and MPLW515L/K mutations. *Leukemia* **21**, 1658-1668 (2007).
12. Walters, D.K. et al. Activating alleles of JAK3 in acute megakaryoblastic leukemia. *Cancer cell* **10**, 65-75 (2006).
13. Griffin, J.H., Leung, J., Bruner, R.J., Caligiuri, M.A. & Briesewitz, R. Discovery of a fusion kinase in EOL-1 cells and idiopathic hypereosinophilic syndrome. *Proceedings of the National Academy of Sciences of the United States of America* **100**, 7830-7835 (2003).
14. Zheng, R. et al. FLT3 ligand causes autocrine signaling in acute myeloid leukemia cells. *Blood* **103**, 267-274 (2004).
15. Milella, M. et al. Therapeutic targeting of the MEK/MAPK signal transduction module in acute myeloid leukemia. *The Journal of clinical investigation* **108**, 851-859 (2001).
16. Sanda, T. et al. TYK2-STAT1-BCL2 pathway dependence in T-cell acute lymphoblastic leukemia. *Cancer discovery* **3**, 564-577 (2013).
17. Wilson, T.R. et al. Widespread potential for growth-factor-driven resistance to anticancer kinase inhibitors. *Nature* **487**, 505-509 (2012).
18. Shinomiya, N. et al. RNA interference reveals that ligand-independent met activity is required for tumor cell signaling and survival. *Cancer research* **64**, 7962-7970 (2004).

19. Yoshikawa, M. et al. xCT inhibition depletes CD44v-expressing tumor cells that are resistant to EGFR-targeted therapy in head and neck squamous cell carcinoma. *Cancer research* **73**, 1855-1866 (2013).
20. Wilson, T.R., Lee, D.Y., Berry, L., Shames, D.S. & Settleman, J. Neuregulin-1-mediated autocrine signaling underlies sensitivity to HER2 kinase inhibitors in a subset of human cancers. *Cancer cell* **20**, 158-172 (2011).
21. Drosten, M., Frilling, A., Stiewe, T. & Putzer, B.M. A new therapeutic approach in medullary thyroid cancer treatment: inhibition of oncogenic RET signaling by adenoviral vector-mediated expression of a dominant-negative RET mutant. *Surgery* **132**, 991-997; discussion 997 (2002).
22. Zhang, C., Habets, G. & Bollag, G. Interrogating the kinome. *Nature biotechnology* **29**, 981-983 (2011).

Supplementary Note 1

Calculation of kinase score and kinase rank

Depicted is the stepwise calculation of the kinase score and the kinase rank for a fictive test cell line.
Matrices of five different drugs, comparator cell lines, and kinases are used for the example. Each of the eight numbers on the left side refers to the calculation step described in the "Algorithm" part of the Material and Methods section.

1.)

Calculation of:
"average % living comparator cells"

<u>% living comparator cells after 72 hours inhibitor treatment</u>

	Drug 1	Drug 2	Drug 3	Drug 4	Drug 5
Compar. cell line 1	90%	11%	99%	97%	67%
Compar. cell line 2	16%	73%	23%	3%	99%
Compar. cell line 3	42%	66%	19%	72%	18%
Compar. cell line 4	91%	89%	17%	5%	55%
Compar. cell line 5	72%	3%	93%	1%	52%
	$\bar{}$, average				
**"average % living comparator cells"**	62%	48%	50%	36%	58%

2.)

Calculation of:
"relative % apoptosis"

	Drug 1	Drug 2	Drug 3	Drug 4	Drug 5
"average % living comparator cells"	62%	48%	50%	36%	58%
	-, minus				
"% living test cells"	29%	5%	73%	81%	14%
	=, equals				
**"relative % apoptosis"**	33%	43%	- 23%	- 45%	44%

3.)

Calculation of:
“Score I” and
“Kinase rank I” by formula #1

<u>% enzymatic inhibition</u>

	Drug 1	Drug 2	Drug 3	Drug 4	Drug 5
Kinase A	32%	41%		7%	33%
Kinase B		22%	11%	23%	72%
Kinase C	91%	58%	12%	37%	
Kinase D		75%	8%		
Kinase E	13%	7%		51%	24%

< 5% enzymatic inhibition

	Drug 1	Drug 2	Drug 3	Drug 4	Drug 5
Kinase A	32%	41%		7%	33%
	x, times				
“relative % apoptosis”	33%	43%	- 23%	- 45%	44%
	=, equals				
	1056	1763		-315	1452

Number of drugs inhibiting kinase A to more than 5%: 4

Σ =, sum equals 3956

÷ divide by

4

= equals

“Score I” of kinase A : 989

Calculate *Score I* by formula #1 for all kinases

	Score I
Kinase A	989
Kinase B	707
Kinase C	889
Kinase D	1014
Kinase E	-127

Rank all kinases based on “score I” from high to low

Formula #1

	Score I	Kinase rank I
Kinase A	989	2
Kinase B	707	4
Kinase C	889	3
Kinase D	1014	1
Kinase E	-127	5

4.)

Calculation of:
"Score I" and
"Kinase rank I" by formula #2

<u>% enzymatic inhibition</u>

	Drug 1	Drug 2	Drug 3	Drug 4	Drug 5
Kinase A	32%	41%	1%	7%	33%
Kinase B	1%	22%	11%	23%	72%
Kinase C	91%	58%	12%	37%	4%
Kinase D	2%	75%	8%	4%	1%
Kinase E	13%	7%	3%	51%	24%

	Drug 1	Drug 2	Drug 3	Drug 4	Drug 5		
Kinase A	32%	41%	1%	7%	33%	Σ =, sum equals	114
	x, times						
"relative % apoptosis"	33%	43%	- 23%	- 45%	44%		
	=, equals						
	1056	1763	-23	-315	1452	Σ =, sum equals	3933

3933
÷
divide by
114
=
equals

"Score I" of kinase A :	35

Calculate "score I" by formula #2 for all kinases

	Score I
Kinase A	35
Kinase B	22
Kinase C	18
Kinase D	33
Kinase E	-6

Rank all kinases based on *Score I* from high to low

Formula #2

	Score I	Kinase rank I
Kinase A	35	1
Kinase B	22	3
Kinase C	18	4
Kinase D	33	2
Kinase E	-6	5

5.)

Calculation of:
“Score II” and “Kinase rank II”

(only calculation steps performed with “Score I” and “Kinase rank I” by formula #1 are shown.
All following calculations have also to be made with “Score I” and “Kinase rank I” by formula #2.)

	**Test cell line**		**Compar. cell line 1**		**Score II (vs. comparator cell line 1)**
	Score I		Score I		
Kinase A	989	-, minus	279	=, equals	710
Kinase B	707		601		106
Kinase C	889		1277		-388
Kinase D	1014		814		200
Kinase E	-127		-719		592

Rank all kinases based on “score II” from high to low

	Score II (vs. comparator cell line 1)	**Kinase rank II (vs. comparator cell line 1)**
Kinase A	710	1
Kinase B	106	4
Kinase C	-388	5
Kinase D	200	3
Kinase E	592	2

Repeat calculation step 5.) with all comparator cell lines

6.)

Calculation of:
"Score III" and "Kinase rank III"

	Kinase rank I	x 3 =, times 3 equals		+, plus	Kinase rank II (vs. compar. cell line 1)	=, equals	**Score III (vs. comparator cell line 1)**
Kinase A	2		6		1		7
Kinase B	4		12		4		16
Kinase C	3		9		5		14
Kinase D	1		3		3		6
Kinase E	5		15		2		17

Rank all kinases based on "score III" from low to high

	Score III (vs. comparator cell line 1)	**Kinase rank III (vs. comparator cell line 1)**
Kinase A	7	2
Kinase B	16	4
Kinase C	14	3
Kinase D	6	1
Kinase E	17	5

Repeat calculation step 6.) with all comparator cell lines

7.)

Calculation of:
"Score IV"

	Kinase rank III (vs. compar. cell line 1)	Kinase rank III (vs. compar. cell line 2)	Kinase rank III (vs. compar. cell line 3)	Kinase rank III (vs. compar. cell line 4)	Kinase rank III (vs. compar. cell line 5)		**Score IV**
Kinase A	2	2	5	1	1	∑, average	2.2
Kinase B	4	5	1	5	3		3.6
Kinase C	3	1	2	4	2		2.4
Kinase D	1	3	3	2	4		2.6
Kinase E	5	4	4	3	5		4.2

8.)

Calculation of:
Kinase score
and final "Kinase rank"

(Score IV calculated by formula #1 and formula #2 are needed for this calculation step.)

	Score IV (as calculated by formula #1)	Score IV (as calculated by formula #2)
Kinase A	2.2	1.4
Kinase B	3.6	3.1
Kinase C	2.4	3.7
Kinase D	2.6	2.9
Kinase E	4.2	3.8

Compare both values for each kinase and insert the lower value in a new table

**Kinase score**
1.4
3.1
2.4
2.6
3.8

Rank all kinases based on their kinase scores from low to high

	Kinase score	**Kinase rank**
Kinase A	1.4	1
Kinase B	3.1	4
Kinase C	2.4	2
Kinase D	2.6	3
Kinase E	3.8	5

Supplementary Note 2

A) Tutorial for the Excel- program used to calculate the kinase rank of cell lines

The Excel-file for the calculation of the kinase rank can be found in the Supplementary Data Set 3. The algorithm described above is implemented by this program.

The program is colour coded throughout the whole file:

- Green sheets or rows: Input needed
- Orange sheets or rows: Input optional
- Red sheets or rows: Important information/output
- Grey sheets: protected sheets; no action needed

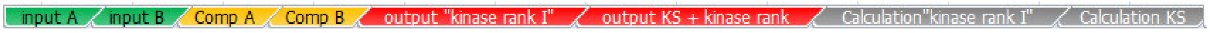

To explain in five steps how the program is used, the calculation of the kinase rank for the human cell line HEL is described.

1.) **Open Supplementary Data Set 4 for dose response data**

2.) **Open Supplementary Data Set 3.**

Go to sheet input A **.**

Insert dose response data (% living cells after 72 hours of inhibitor treatment) of HEL cells where stated (row 8).

3.) **Go to sheet** Comp A **.**

Copy dose response data from all hematopoietic comparator cell lines (panel A) except HEL (see Supplementary Fig. 1c for a complete list of hematopoietic comparator cell lines).

Go to sheet input A **.**

Paste data into the green indicated rows where stated (row 10).

4.) **Go to sheet** Comp B **.**

Copy kinase, score and rank of "kinase rank I" (Algo #1 and #2, row 3 – 564) of the same comparator cell lines as the one's used in step 3.

Go to sheet input B **.**

Paste data into the green empty columns (column B - KO).

5.) **Go to** output KS + kinase rank **.**

The kinase score (KS) and final kinase rank of HEL cells is shown on this sheet.

B) Expansion of the existing comparator cell line library

1. **Go to sheet** Comp A **.**
   Here one can find the % living comparator cells after 72 hours of inhibitor treatment of 44 distinct comparator cell lines. In the orange section (row 52 – 248) one can include additional data derived from new comparator cell lines to expand this “comparator cell line library”. If including dose response data of new comparator cell lines with known kinase addiction delete each value representing the response to a drug that inhibits the addicted kinase to more than 40% (e.g. for the ALK dependent cell line SU-DHL1 all responses to drugs inhibiting ALK to more than 40% have been excluded).

2. **Go to sheet** Comp B **.**
   The “kinase rank I” as calculated by algorithm #1 and algorithm #2 (see the "Algorithm" part in Material and Methods) of all 44 comparator cell lines can be found in sheet Comp B
   In the orange section (column ED – UJ) one can include the “kinase rank I” of additional comparator cell lines. To generate the “kinase rank I” for a new comparator cell line, follow the steps 2-3 described in section A) using the new comparator cell line instead of HEL. The “kinase rank I” for the new comparator cell line will be shown in sheet output "kinase rank I"

   If including new comparator cell lines with known kinase addiction, it is advised to delete from “kinase rank I” all addicted kinases (name+score+rank) and all kinases belonging to the same subfamily (e.g. for the BCR-ABL1 dependent cell line K562 the kinases ABL1 as well as ABL2 have been excluded). Further exclude all kinases with a similar drug selectivity profile as the addicted kinase (the hierarchical clustering of kinases presented in Supp. Figure 2 of Anastassiadis et al. have been used to define kinases with a similar drug selectivity profile. E.g. for K562 cells, besides ABL1 and ABL2, the kinases LynB and PDGFRα have been excluded).

Supplementary Material and Methods

Cell lines used for KISMET

Cell lines were maintained at 37 °C in a humidified atmosphere at 5% CO_2 and grown in RPMI 1640 or DMEM/F12 growth media (GIBCO) supplemented with 10% fetal bovine serum (GIBCO), 50 units ml^{-1} penicillin and 50 µg ml^{-1} streptomycin (SIGMA). 1ng ml^{-1} murine IL-3 (Peptrotech) was added to the growth media of parental Ba/F3 and 32D cells. Human cell lines were derived from the Wellcome Trust Sanger Institute (Cambridge) and have been authenticated by 'fingerprinting' using a panel of 94 single nucleotide polymorphisms and 16 short tandem repeats (STRs) and are genetically unique. Where available the STR profiles have been matched to those published by the cell line repositories. Cells have been used for the experiments within two month after thawing the cells.

Inhibitors

Inhibitors were purchased from Calbiochem (Cat.# 539743-1EA, InhibitorSelect™ 384-Well Protein Kinase Inhibitor Library I: inhibitor# 1-147, #149-153; see Supp. Data Set 1), Selleckchem (Cat.# L1200, Kinase Inhibitor Screening Library: inhibitor# 148, #154-202), AdooQ (PD0325901), Stratech Scientific (MG-132), and Sigma-Aldrich (Cyclohexamide). Stock solutions (10 mM) of kinase inhibitors were prepared in DMSO and diluted in cell culture medium to indicated final concentrations.

Statistics

Cell viability assays of cell lines treated with the kinase inhibitor library were performed in duplicates at two different time points and in four technical replicates for the calculation of the IC_{50} value of the individual inhibitors. Differences were assessed for statistical significance by an unpaired two-tailed t test using GraphPad Prism software.

Single-dose response values

All 202 inhibitors used for KISMET were diluted in RPMI (10% FCS and 1% pen./strep. added) to a final concentration of 10 µM and pipetted into three 96-well sterile tissue culture plates with a

clear bottom (32 µl/well, Greiner bio-one). Sixteen wells per plate (row 1 and 12) contained RPMI only which served as controls. All plates were stored at -80˚C until used.

Dilution series with the individual cell lines were made prior to the screen to identify an appropriate cell density per well. Test cells were incubated with inhibitors (48 µl cells + 32 µl inhibitors) at 37˚C and 5% CO_2. All cell lines have been analysed in duplicates at two different time points. A cell-viability assay using CellTiter-Glo (Promega) was performed to measure the amount of living cells after 72 hours. CellTiter-Glo was diluted 1:5 in sterile H_2O, 80 µl/well were added to the cells and incubated at RT for 20 min. Cell viability was measured in a standard luminometer (GloMax@ 96, Promega) with an integration time of 1 sec/well.

The "% living cells" per drug has been calculated as the average relative luminescence unit (RLU) of inhibitor treated cells divided by the RLU of untreated control cells, and multiplied by 100.

Combination of two off-target data sets

172 inhibitors as published by Anastassiadis et al. and 30 inhibitors as published by Davis et al. have been used for KISMET. Both groups used different assays to generate their off-target data set. Of the 1513 pairs of kinases and kinase inhibitors that have been common to both studies, the binding affinities measured by Davis et al are in relatively good agreement with the remaining kinase activity in a single-dose inhibitor (500 nM) measured by Anastassiadis et al.[3] This comparison of 1513 pairs has been used to transform each dissociation constant (K_d) from Davis at al into "% kinase inhibition" value (as measured by Anastassiadis et al). The table that has been generated to transform each single K_d is shown in Supplementary Table 3. This enabled us to use both datasets as a single measure.

Selection of kinases used for KISMET

Although an off-target profile of more than 400 kinases has been published,[1,2] we included only kinases for which the enzymatic inhibition has been shown for the majority of the 202 inhibitors. Therefore we have excluded kinases published only by Davis et al.[2] Of the remaining 300

kinases we included only those with a strong affinity to our 202 drugs to increase the reliability of the output. To this end we calculated the "sum of inhibition" (SOI) for each kinase and all 202 inhibitors using following formula:

$$\sum_{i=1}^{202} \%\ enzymatic\ inhibition\ of\ kinase\ X\ by\ inhibitor\ i$$

The average SOI of all 300 kinases is 1997 ± 1088 with FLT3 having the highest SOI (7404) and DMPK the lowest SOI (298). Including kinases with a SOI < 600 caused the KISMET algorithm to output a biased ranking. Several kinases having a SOI < 600 got ranked in a top position in cell lines with known kinase addiction although not enough off-target information is provided by the 202 inhibitors to support this ranking. Therefore we excluded 21 kinases having a SOI < 600 (Supplementary Fig. 5). The remaining 279 kinases were used for KISMET.

Algorithm for the calculation of the kinase rank

Supplementary Note 1 shows the stepwise calculation of the kinase rank presented below using matrices of five inhibitors, five kinases, and five comparator cell lines. The Excel program which has been used for the matrices calculations to generate the kinase ranks can be found in Supplementary Data Set 3 and a tutorial explaining its usage is described in the Supplementary Note 2.

To make the output more robust the algorithm used to calculate the kinase score (KS) and kinase rank integrates data derived from comparator cell lines. The more comparator cell lines used, and the more heterogeneous their composition is, the more reliable the output. The test cell line has been excluded if present in the comparator panel. If comparator cell lines with known kinase addiction are used, all drug-response values ("% living cells") derived from drugs that inhibit the addicted kinase to more than 40% are excluded.

1.) Calculate the "average % living comparator cells" upon treatment with drug 1 as follows:

“average % living comparator cells” =

$$\frac{\sum_{i=1}^{n} \% \; living \; cells \; of \; \text{comparator} \; cell \; line \; i \; upon \; treatment \; with \; drug \; 1}{n}$$

whereby n = the number of comparator cell lines.

Calculate the “average % living comparator cells “ for all 202 drugs.

2.) Calculate the *“relative % cell death”* of your test cells induced by drug 1 as follows:

“relative % cell death” =

(” average % living comparator *cells” upon treatment with drug 1) – (% living test cells upon treatment with drug 1)*

Calculate the “relative % cell death” for all 202 drugs.

“Kinase rank I”

This part of the algorithm calculates a “raw” kinase rank based on the response of the test cell line to each of the 202 kinase inhibitors. The raw kinase rank represents a preliminary list of kinases ranked by their importance for the survival/proliferation of the test cells. Two individual formulas are used for its calculation:

3.) Formula 1:

Exclude all off-target values (% inhibition of kinase activity) that are < 5%.

“Score I” of kinase A =

$$\frac{\sum_{i=1}^{\mathrm{n}} \% \; inhibition \; of \; kinase \; A \; by \; drug \; i \times \text{"}relative \; \% \; apoptosis\text{"} \; induced \; by \; drug \; i}{n}$$

whereby n = number of drugs inhibiting kinase A to more than 5%

Calculate “Score I” for all 279 kinases.

Rank all kinases based on their "Score I" from high to low (highest score = rank #1, lowest score = rank #279) to generate "Kinase rank I".

4.) Formula 2:

"Score I" of kinase A =

$$\frac{\sum_{i=1}^{202} \%\ inhibition\ of\ kinase\ A\ by\ drug\ i \times "relative\ \%\ apoptosis"\ induced\ by\ drug\ i}{\sum_{i=1}^{202} \%\ inhibition\ of\ kinase\ A\ by\ drug\ i}$$

Repeat this step with all 279 kinases.

Rank all kinases based on their "score I" from high to low to generate "kinase rank I".

All following calculation steps have to be done separately with "kinase rank I" as calculated by formula 1 as well as calculated by formula 2.

"Kinase rank II "

If using comparator cell lines with known kinase addiction exclude from "kinase rank I" the addicted kinase ("score I" + rank) as well as all kinases belonging to the same kinase family and kinases showing a similar drug selectivity profile as the addicted kinase (the hierarchical clustering of kinases presented in Supplementary Fig. 2 of Anastassiadis et al. have been used to define kinases with a similar drug selectivity profile). E.g. when using the JAK2 addicted cell line HEL as comparator cell line exclude from its "kinase rank I" the kinase JAK2, the subfamily members JAK1, JAK3 and TYK2 as well as MEKK2 and MEKK3 since they share similar drug selectivity profiles as JAK2.

5.) "Score II" of *kinase A* =

$$("score\ I"\ of\ kinase\ A\ of\ test\ cell\ line) - ("score\ I"\ of\ kinase\ A\ of\ assistant\ cell\ line\ 1)$$

Repeat this step with all 279 kinases.

Rank all kinases based on their “score II” from high to low to generate “kinase rank II” of the test cell line versus comparator cell line 1.

Repeat this step with all comparator cell lines.

“Kinase rank III”

The calculations for “score III” combine kinase rank I and II and shift the weight of the final kinase score KS to kinase rank I.

6.) “Score III” of kinase A =

3 x “kinase rank I” of kinase A + 1 x “kinase rank II” of kinase A versus comparator *cell line 1*

Repeat this step with all 279 kinases.

Rank all kinases based on their “score III” from low to high to generate “kinase rank III” based on comparator cell line 1.

Repeat this calculation with all comparator cell lines.

Kinase score (KS) and final kinase rank

Calculate the “score IV” for kinase A as follows:

7.) “Score IV” *of kinase A =*

$$\frac{\sum_{i=1}^{n} \text{"}kinase\ rank\ III\text{"}\ of\ kinase\ A\ based\ on\ \text{comparator}\ cell\ line\ i}{n}$$

whereby n = the number of comparator cell lines.

Repeat this step with all 279 kinases.

8.) The final kinase score KS and kinase rank of kinase A is calculated as follows:

X = “score IV” of kinase A by formula 1

Y = “score IV” of kinase A by formula 2

If X < Y then KS of kinase A = X

If X >Y then KS of kinase A = Y

Repeat this step with all 279 kinases.

Rank all kinases based on their KS from low to high to get the final kinase rank.
